# Microfluidic Fabrication of Natural Polymer-Based Scaffolds for Tissue Engineering Applications: A Review

**DOI:** 10.3390/biomimetics8010074

**Published:** 2023-02-09

**Authors:** Elisabetta Rosellini, Maria Grazia Cascone

**Affiliations:** Department of Civil and Industrial Engineering, University of Pisa, Largo Lucio Lazzarino, 56122 Pisa, Italy

**Keywords:** microparticles, microfibers, alginate, gelatin, stem cells

## Abstract

Natural polymers, thanks to their intrinsic biocompatibility and biomimicry, have been largely investigated as scaffold materials for tissue engineering applications. Traditional scaffold fabrication methods present several limitations, such as the use of organic solvents, the obtainment of a non-homogeneous structure, the variability in pore size and the lack of pore interconnectivity. These drawbacks can be overcome using innovative and more advanced production techniques based on the use of microfluidic platforms. Droplet microfluidics and microfluidic spinning techniques have recently found applications in the field of tissue engineering to produce microparticles and microfibers that can be used as scaffolds or as building blocks for three-dimensional structures. Compared to standard fabrication technologies, microfluidics-based ones offer several advantages, such as the possibility of obtaining particles and fibers with uniform dimensions. Thus, scaffolds with extremely precise geometry, pore distribution, pore interconnectivity and a uniform pores size can be obtained. Microfluidics can also represent a cheaper manufacturing technique. In this review, the microfluidic fabrication of microparticles, microfibers and three-dimensional scaffolds based on natural polymers will be illustrated. An overview of their applications in different tissue engineering fields will also be provided.

## 1. Introduction

Tissue engineering is an interdisciplinary field that aims to regenerate diseased tissues. The traditional tissue engineering paradigm is based on the combination of three key elements: cells, scaffolds and signals. The scaffold is a biodegradable structure that offers temporary support to the cells during the growth of the new tissue [1].

Both natural [2] and synthetic polymers [3] have been widely investigated as scaffold materials. Natural polymers have certain advantages over synthetic polymers, as they are not only biocompatible and biodegradable but also structurally and compositionally similar to the natural extracellular matrix (ECM).

Such materials have been used to produce scaffolds using various fabrication strategies. Traditional fabrication techniques include bulk emulsion for the fabrication of micro- and nanoparticles; dry spinning, wet spinning and electrospinning for the fabrication of fibers; and freeze-drying, gas foaming and salt leaching for the fabrication of three-dimensional porous scaffolds. However, all of these methods present limitations, such as the use of organic solvents, the obtainment of a non-homogeneous structure, the variability in pore size and the lack of pore interconnectivity.

Over the past two decades, microfluidics has emerged as a new technology for a range of applications, including molecular analysis, laboratory diagnostics and consumer electronics. More recently, biomedical applications have also been explored, and microfluidics has been proposed as a novel strategy to fabricate scaffolds comprising microfibers, microparticles and building blocks for 3D matrices [4].

Microfluidics involves the manipulation of fluids within platforms having channels with dimensions on the order of tens to hundreds of microns. This permits the production of monodisperse structures with tunable sizes.

In this review, after a brief overview of microfluidics and microfluidic devices, natural polymers and methods for polymer cross-linking, we will focus the attention on the production of natural polymer-based scaffolds by microfluidics. In particular, the microfluidic fabrication of (i) particles, (ii) fibers and (iii) 3D matrices will be reviewed, highlighting the advantages of microfluidic-generated scaffolds over conventional scaffolds. The application of microfluidic scaffolds in different tissue engineering fields will also be illustrated.

## 2. Microfluidics and Microfluidic Devices for Scaffold Fabrication

Microfluidics is a multidisciplinary field involving engineering, physics, chemistry and nanotechnologies, through which small amounts of fluids (usually 10^−9^ to 10^−18^ L) [5] can be manipulated and controlled. It involves the use of microdevices with integrated micro-channels (from ten to hundreds of micrometers of diameter) [6] that transport, mix, process or separates the fluids flowing through the channels. The reduced channel diameters lead to the scaling of many physical laws and basic physics that rule microfluidics, as illustrated in [7].

The first microfluidic device was developed in the 1970s, but it took almost 20 years for microfluidics to come to the fore.

In recent years, microfluidic devices have been widely used in biomedical applications due to the major control of the fluids that they offer and their low cost. For example, they have been widely used in the diagnostic [8] and pharmaceutical fields. For the latter, in vitro models, that replicate organ functions and diseases on a microchip [9,10] in order to test new drugs, have been developed. The aim of these models, called “organ-on-chip”, is to increase the predictability of a new drug efficacy before animal testing (possibly even replacing animal trials) and human clinical trials.

More recently, microfluidics has also emerged as an innovative technique for the production of scaffolds with tuneable morphological properties and compositions in a high-throughput manner.

In particular, droplet microfluidics and microfluidic spinning techniques were developed during the 21st century [11]. These two methods are employed in the field of tissue engineering in order to obtain microparticles and microfibers, which are used for the production of injectable microparticle-based scaffolds, fibrous scaffolds or porous three-dimensional (3D) scaffolds.

Microfluidic chips can be fabricated through different methods and using a range of different materials, but for the concerns of tissue engineering, polydimethylsiloxane (PDMS) is the most widely used due to its biocompatibility, relatively low cost, ease of manufacture and sterilization. Alongside PDMS, polycarbonate (PC) has been used to fabricate microfluidic devices as well, especially in recent years. In order to enclose the microchannels, which are usually two/three for the inputs and one for the output, PDMS is bonded to glass slides and treated to make the surfaces chemically inert.

A chip for the production of particles usually has a flow-focusing layout of the junction with input microchannels connected to reservoirs or micropipettes containing different immiscible solutions whose flow is laminar due to channel size [12]. If the aim is to produce empty polymer microparticles, the side input channels contain a solution of the polymer, which will be used to form the particles, while the middle one contains a gas phase, whose pressure represents an important parameter that can be regulated in order to achieve a specific particle diameter. Alternatively, an oil/surfactant solution is used in the side channels and the polymer one in the middle to obtain solid polymer microparticles. Both empty and solid polymeric microparticles are generally produced at a rate of hundreds or thousands per second in a highly reproducible manner [6].

In microfluidic spinning, the chip is quite similar, but the output channel is generally formed by the union of three inputs, containing two different solutions: a polymer solution in the middle channel and a sheath fluid in the side ones. The polymer fibers, with smooth and round surfaces, are formed along the output channel, while the sheath flow passes through the polymer solution and is then collected.

The dimensions of both microfibers and microparticles can be precisely defined by setting appropriate parameters, such as flow rate, gas pressure and the concentration of the solutions. In general, a higher speed of the oil/surfactant solution or of the sheath fluid leads to a smaller diameter of particles or fibers, respectively. On the contrary, as the flow rate of the polymer solution increases, the diameter increases as well [13,14,15].

Further details on the microfluidic production of polymeric microparticles and microfibers are provided in Section 4.1 and Section 4.2, respectively.

## 3. Natural Polymers Used for Microfluidic Scaffolds

The aim of a tissue engineering scaffold is to temporally replace the native ECM, offering mechanical support to cells and tuning cell behavior. The ECM mainly consists of collagen, elastin, glycoproteins and polysaccharides; therefore, natural polymers, including proteins and polysaccharides, have been largely investigated for the production of scaffolds using several procedures, including microfluidic techniques [16]. A brief overview of the natural polymers most frequently used to produce scaffolds by microfluidics will be provided in the next sections.

### 3.1. Polysaccharides

Starting from the second half of the last century, polysaccharide materials have been widely used for their remarkable properties in the field of biomedical sciences [17]. Natural polysaccharides are considered to be superior to other polymers due to their ease in tailoring, biocompatibility, bioactivity, homogeneity, bio-adhesive properties and ability to mimic the ECM microenvironment. They perform different physiological functions and, therefore, may offer a variety of potential applications in the fields of tissue engineering and regenerative medicine.

Polysaccharides are naturally found in plants, microbes and animal sources and are, therefore, widely distributed in nature [18,19]. The main characteristics of two of the natural polysaccharides mainly used for the production of scaffolds, alginate and chitosan, will be described below.

#### 3.1.1. Alginate

Alginate, the monovalent form of alginic acid, is one of the most widely explored seaweed polysaccharides [20]. As a U.S. Food and Drug Administration (FDA)-approved polymer, alginate has become one of the most important biomaterials for different applications in various fields, including regeneration medicine [21].

Alginate is a linear block copolymer, composed of β-(1→4)-linked D-mannuronic acid (M block) and α-L-guluronic acid (G block) units, arranged with varying proportions of GG, MM and GM blocks [22]. The proportions of M and G residues and the lengths of the blocks can vary considerably depending on the source of the alginate. The biological and physical properties of alginates in aqueous media [23] depend not only on the M/G ratio but also on the distribution of M and G units along the chains. In addition, the molecular weight (MW) of alginate influences the degradation rate and mechanical properties of alginate-based biomaterials.

Alginate undergoes ionotropic gelation in the presence of divalent cations, such as Ca^2+^ and adjacent G residues, following the model of an egg box [22].

Alginate-based biomaterials can be utilized as drug delivery systems [24] and cell carriers [25] for tissue engineering. Alginate can be easily modified via chemical and physical reactions to obtain derivatives having various structures, properties, functions and applications. In particular, in combination with other biomaterials, immobilization of specific ligands, such as peptide and sugar molecules, and cross-linking treatments can be used to fine-tune alginate structure and properties, such as biodegradability, mechanical strength, gelation property and cell affinity [21].

#### 3.1.2. Chitosan

Chitosan, the N-deacetylated derivative of chitin, which forms the exoskeleton of crustacean shells, belongs to the polysaccharide family and has structural similarities to glycosaminoglycans. Chitosan has been widely investigated as a natural biomaterial for many biomedical applications, including tissue engineering, due to its biocompatibility, biodegradability and antimicrobial properties [26,27,28]. Chitosan can be molded into porous scaffolds through various simple procedures such as lyophilization, gas foaming and electrospinning, and it can form blends with various natural and synthetic polymers, thereby generating scaffolds with desired properties. The cationic nature of chitosan aids in the formation of polyelectrolyte complexes with a wide range of anionic glycosaminoglycans (GAGs), including heparin and chondroitin sulphate. These chitosan–GAGs complexes may modulate the activity of several growth factors and cytokines and have been demonstrated to be promising in bone and cartilage regeneration. In addition, chitosan is a biodegradable polymer, and its degradation rate can be modulated easily by the degree of deacetylation to meet the regeneration requirements of different tissues. Finally, the availability of functional groups on chitosan provides an opportunity for the conjugation of biologically active molecules (such as adhesive proteins and peptides), thereby supporting cell growth, differentiation and proliferation.

### 3.2. Proteins

Proteins are natural biodegradable materials, and their use in the production of scaffolds for tissue engineering has received considerable attention. Proteins have excellent biocompatibility and biodegradability, and their degradation products, amino acids, are the basic components of life and, therefore, can be resorbed as nutrients. In addition, some of them induce minimal tissue inflammatory responses, are available on a large scale and have low costs [28]. Fibrous proteins, such as collagen, are particularly interesting for the production of scaffolds. They are characterized by highly repetitive amino acid sequences that primarily provide mechanical and architectural functions in nature. The repetitive primary sequences result in the formation of relatively homogeneous secondary structures via self-assembly, leading to the formation of fibrous structural materials [29].

The main characteristics of the fibrous proteins that are more frequently used for the microfluidic production of scaffolds, including collagen, gelatin and modified gelatin, will be described below.

#### 3.2.1. Collagen

Collagen is one of the most studied biomolecules of the ECM due to its presence in all connective tissues [30]. This fibrous protein is the major component of skin and bone and represents approximately 25% of the total dry weight of mammals. Twenty-nine distinct collagen types are currently known, and all of them display a typical triple helix structure. Type I collagen is currently the gold standard in the field of tissue engineering.

Collagen biocompatibility and possible degradation by human collagenases are responsible for the widespread use of this material. Cell–collagen interactions are naturally favored, as cell receptors recognize specific peptide sequences within collagen molecules.

Collagen-based scaffolds can undergo different cross-linking treatments (chemical, physical, enzymatic) to control their mechanical and degradation behaviors.

#### 3.2.2. Gelatin

Gelatin is a natural biopolymer derived either by partial acid (gelatin type A) or alkaline hydrolysis (gelatin type B) of collagen from skin, bone and tendon [31]. Gelatin is biocompatible, biodegradable and exhibits low antigenicity. Moreover, it is commercially available at a low cost. It does not produce harmful byproducts upon enzymatic degradation and contains peptide sequences that modulate cell adhesion. It also has a high number of accessible functional groups for modification by cross-linking or the coupling of bioactive molecules.

Thanks to these properties, gelatin has been widely investigated for its use as a scaffold material [31].

Several techniques have been employed to fabricate gelatin scaffolds, such as electrospinning, phase separation, porogen leaching and self-assembly. One of the main limitations of gelatin-based scaffolds is their poor mechanical properties, especially under wet conditions, which can be enhanced by blending them with synthetic polymers and through cross-linking treatments [31].

#### 3.2.3. Gelatin Methacryloyl

Gelatin methacryloyl (GelMA) is a versatile biomaterial with tunable physicochemical properties and promising remarkable compatibility for a wide spectrum of applications.

The introduction of methacryloyl substituent groups confers to gelatin the property of photocrosslinking with the assistance of a photoinitiator and exposure to light due to the photopolymerization of the methacryloyl substituents. This polymerization can take place under mild conditions (room temperature, neutral pH, aqueous environments, etc.) and allows for the temporal and spatial control of the reaction.

The chemical modification of gelatin using methacrylic anhydride (MA) generally involves less than 5% of the amino acid residues in molar ratio, which implies that most of the functional amino acid motifs (such as adhesive motifs and enzyme-degradable motifs) will not be significantly influenced [32]. The biocompatibility of GelMA, commercially available from multiple vendors, has been demonstrated in multiple studies involving materials that were processed using UV stereolithography and two-photon polymerization [33].

In addition to GelMA, it is worth mentioning that another form of modified gelatin, which is gelatin with increased phenolic hydroxyl content (GelPh), is receiving interest [34].

### 3.3. Cross-Linking Strategies for Natural Polymers

Several strategies have been devised for the cross-linking of the natural polymers used in biomedical applications due to the fact that they possess insufficient stability in physiological aqueous environments. Generally, cross-linking reactions improve stability by using interconnecting molecules; however, cross-linking could cause reduced degradability and the lower availability of functional groups, and, in the case of cell-laden scaffolds, this could negatively impact cell vitality. Thus, the selection of an appropriate cross-linking approach is critical for the successful application of natural polymer-based scaffolds. Cross-linking methods include the following:

- Ionic cross-linking: addition of divalent cations to the natural polymer solution (e.g., alginate) enables the rapid formation of a hydrogel [35];
- Temperature-induced gelation and freezing: the gelation method is applicable to scaffolds produced using natural polymers, such as collagen and gelatin, which can be transformed into hydrogels by simply changing the temperature and, therefore, forming hydrophobic interactions between the molecules. The freezing method relies on the fact that some hydrocarbon molecules possess melting points at or above room temperature, so working below that temperature allows the formation of hydrogen bonding between the molecules, cross-linking the material [36].
- Chemical cross-linking: the gelation of biopolymers is enabled by chemical linkers that form bonds between two functional groups of different molecules. For example, glutaraldehyde is used as a chemical cross-linker [37], which binds to amino groups from lysine and amide groups from asparagine or glutamine residues in two different collagen or gelatin molecules;
- Photo-cross-linking and enzymatic cross-linking: these methods take advantage of a photo-initiator or an enzyme to form bonds between the molecules. These approaches are attractive for the in situ gelation of scaffolds, due to rapid gelation (normally no more than 10 min), through the formation of strong covalent bonding at room temperature and under mild conditions [38].

When a microfluidic device is used, all of these cross-linking approaches can be applied either inside or outside of the device. However, for tissue engineering applications, cross-linking inside the device is widely preferred as it allows better control over the shape and the exposure time of chemical cross-linkers or UV radiations, which can affect the viability of the cells eventually laden in the scaffolds. In addition, a cross-linking process conducted inside the microchannels allows for better control over the degree of cross-linking of the produced material.

## 4. Scaffold Production Using Microfluidics

### 4.1. Microparticles

Polymeric microparticles have received significant interest for use as scaffolds for tissue engineering [39]. Cells can be seeded and grown over a particle surface or can be encapsulated within particles. The main advantage of microparticles is their high surface-to-volume ratio, promoting nutrient transfer and improve cell–matrix interactions. Precise control over particle size and size distribution is important, as these two parameters can influence cell phenotype. Moreover, the microparticle diameter should fall within the 60–200 µm size to promote high cell viability and proliferation. In fact, a diameter below 60 is too small to promote cell contact and proliferation; on the other hand, a diameter above 200 could hinder adequate nutrient and oxygen transport across the polymeric matrix.

The production of MPs is a process that consists of at least two steps: droplet formation and subsequent cross-linking to enhance their structural stability. Prior to the development of microfluidics, polymeric microparticles were mainly produced using traditional methods, such as the following:

- Droplet breakup from an emulsion using shear or impact stresses generated by the agitation of two immiscible fluids and subsequent cross-linking;
- Coacervation, which is based on the separation of an aqueous polymeric solution into two miscible liquid phases: a dense coacervate phase and a dilute equilibrium phase [40];
- Spray drying, consisting of the transformation of fluid materials into dried particles, taking advantage of a gaseous hot-drying medium [41].

However, the main limitation is that all of these methods do not allow for precise control over particle size and size distribution, leading to a polydisperse particle distribution.

A promising alternative to traditional emulsions is microfluidics, which can be used to rapidly generate particles with tunable sizes and a polydispersity index below 2–3% by simply manipulating and controlling the flow of multiple immiscible liquids. A common strategy to control particle diameter is to vary the flow rate ratio between the dispersed phase and the continuous one. Microfluidics also offers easy control over the shape and morphology of the resulting particles.

An overview of the microparticles obtained through microfluidic techniques and used as scaffolds in tissue engineering is provided in Table 1.

#### Microparticles Fabrication Using Microfluidic Devices

**Figure 1:**
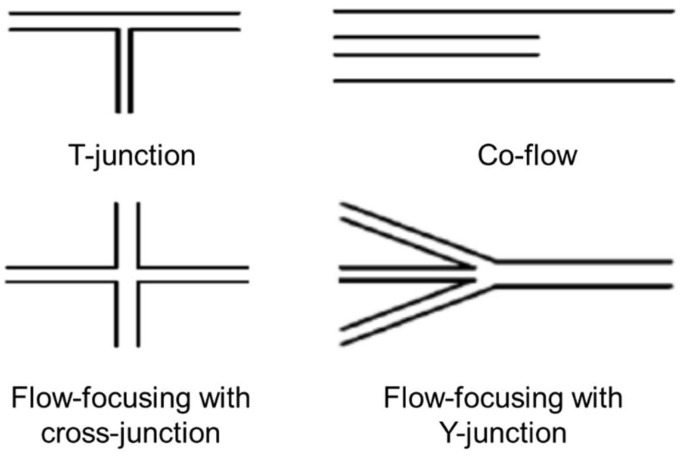
Microfluidic geometries typically used for microparticles fabrication.

The typical microfluidic geometries used in generating microparticles are T-junction, co-flow and flow-focusing, with cross-junction or Y-junction configurations (Figure 1).

In the T-junction, the dispersed phase is sheared in a T-shaped junction, which has an angle θ (0° < θ ≤ 180°) between the dispersed and the continuous phase channels. The generated droplets have a high monodispersity, with a coefficient of variation (CV) typically less than 2%. The size of the droplets generated in a T-junction is generally determined by the channel dimensions.

In the co-flow geometry, also called the coaxial junction, a dispersed phase channel is inserted into and aligned with a continuous phase channel, and the dispersed phase and continuous phase fluids flow in parallel through the channels. In most cases, droplets are formed in a dripping mode and have a low CV (less than 3%). However, the droplet sizes are typically larger than the diameter of the dispersed phase channel.

Flow-focusing has a focus unit that suddenly shrinks the fluid passageway. Channels can create a cross-junction or a Y-junction. Fluid phases form a hydrodynamic flow that contracts through the focus unit resulting in high fluid-flow rates. This enhances the viscous shear force and enables the formation of droplets with sizes down to a few hundred nanometers [36].

It is important to underline that these typical device geometries can be customized for use in specific applications. For example, a customized microfluidic device was developed for the encapsulation of stem cells within monodisperse alginate–collagen microcapsules without the use of surfactants [45]. In another work, an innovative microfluidic device was developed with the aim of improving the biocompatibility of cell-laden microgels fabricated via a microfluidic flow-focusing device [48]. If cell-laden microgels are produced through a single-emulsion microfluidic flow-focusing device, during droplets generation, some cells are exposed to the oil phase, which can have a cytotoxic effect. In order to solve this issue, a double-emulsion flow-focusing device was developed by Kim et al. [48], in which a secondary aqueous phase containing cells enters the primary aqueous phase, minimizing the exposition of cells to the oil phase. Such a device maintained the typical advantages of microfluidic fabrication of cell-laden microspheres, improving process biocompatibility.

Moreover, in addition to conventional droplet-based microfluidics, centrifugal microfluidic devices have been developed for hydrogel microparticles fabrication. Centrifugal microfluidic devices use centrifugal force to disperse a pre-gel solution and collect hydrogel particles in the cross-linking solution. In general, glass capillaries are fixed to a centrifuge tube, and all of the liquid in the capillaries is dispersed in the collecting bath under centrifugal force. For example, Cheng et al. designed a novel centrifugal microfluidic system to produce both simple and core–shell alginate microparticles [47].

### 4.2. Microfibers

Among the existing scaffolds, 3D fibrous scaffolds have received significant attention as their fibrous networks can efficiently mimic the structure of the natural ECM. In addition, fibers can be collected and processed into complex fibrous networks using conventional textile techniques, such as knitting, weaving or braiding, to create 3D structures with improved structural and mechanical properties.

Polymeric fibers are traditionally produced through spinning processes [52]. These include the following:

- Wet Spinning: in this method, a viscous polymer solution is maintained in a syringe and then extruded through a continuous filament and collected into a coagulation bath, made by a nonsolvent for the polymer. Nonsolvent-induced phase separation transforms liquid polymer streams into solid filaments, that are subsequently wound up with a spool. The diameters of the filaments produced through the use of wet spinning methods are typically 30–200 µm, but can be as small as 4–10 µm;
- Electrospinning: the basic setup for this technique involves a polymeric solution contained in a nozzle-fitted syringe, a pump, a high-voltage power source and a collector. When sufficiently high voltage is applied to the syringe needle, the liquid surface becomes charged. When the electrostatic repulsion is higher than the surface tension, the liquid surface is deformed into a conically shaped structure known as the Taylor cone. Once the Taylor cone is formed, the charged liquid jet is ejected toward the collector, where the fiber is deposited. The diameter of these fibers typically ranges between tens of nanometres to a few micrometers;
- Dry spinning: the polymer solution is collected into a syringe and pushed down in a vertical cylindrical tube. Simultaneously, hot air is blown from the bottom upwards, transforming the polymer solution into a solid thread, which can be rolled with a spool. This method produces micro-sized fibers;
- Melt spinning: the fiber-forming substance is melted and then pumped through a spinneret in an air chamber. The extruded stream cools and solidifies into continuous filaments and is subsequently wound onto spools. This is the most convenient and economical method for polymer microfiber manufacturing at an industrial scale.

Recently, microfluidics has been proposed as a new strategy for the fabrication of fibrous scaffolds. Indeed, traditional spinning methods have some limitations, starting from the fact that most of them make use of high temperatures or harsh solvents and are not adequate for cell encapsulation. Moreover, it is difficult to ensure repeatable results in terms of mechanical and morphological properties as the spinning process depends on many variables. Some spinning processes cannot be used for natural polymers because these are decomposed or degraded by the temperatures necessary for extrusion (i.e., melt spinning). Lastly, spinning processes do not allow for the production of fibers with a layering of different materials or with complex cross-sectional shapes, such as tubular or flat ones.

Most of these problems can be easily overcome using microfluidics as it enables greater control over the final morphology and mechanical properties of the end product, as well as easy cell loading within the fiber structure, to create more complex scaffolds that better mimic the natural ECM.

An overview of the microfluidic fabricated microfibers used as scaffolds for tissue engineering is provided in Table 2.

#### Fiber Fabrication Using Microfluidic Devices

**Figure 2:**
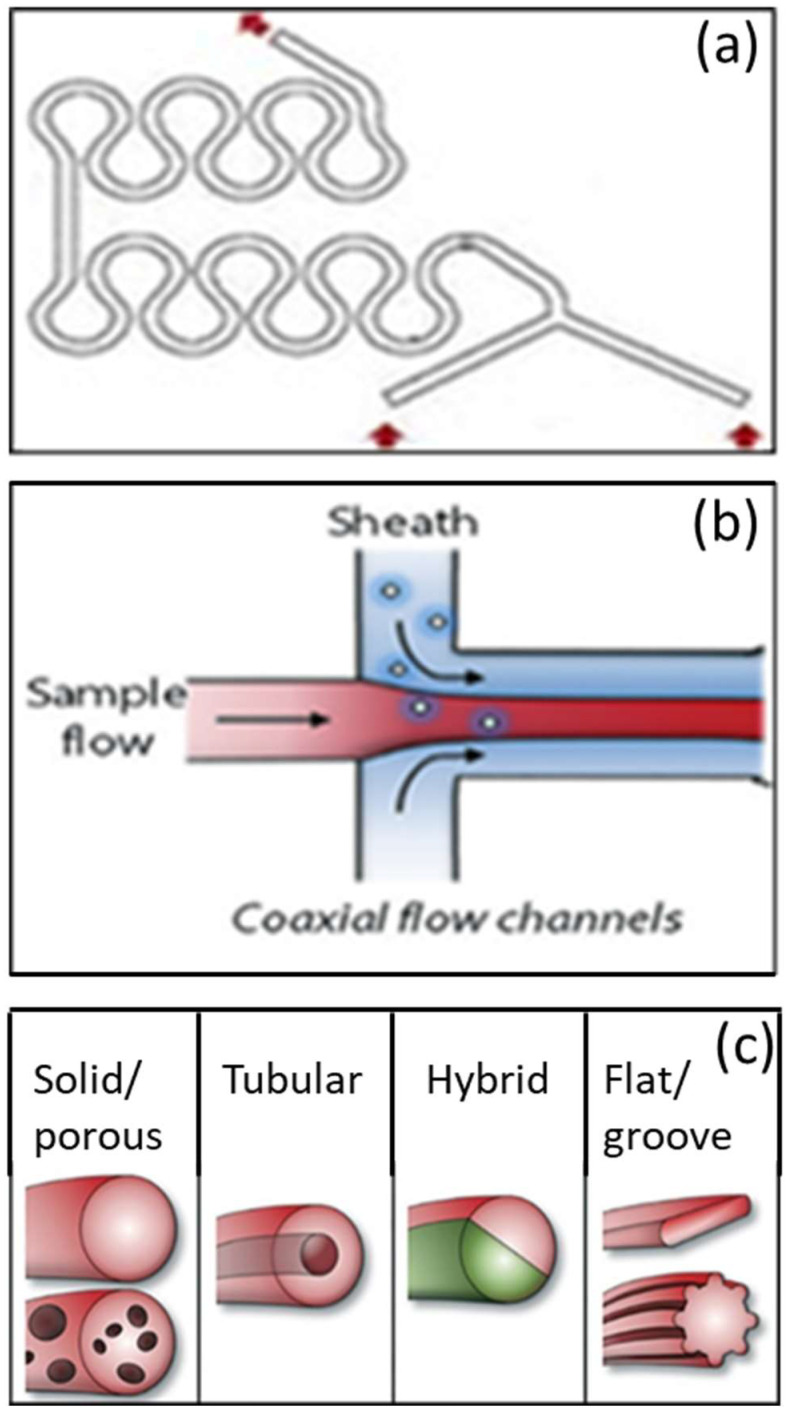
(**a**) Example of a snake-like channel; (**b**) Schematic diagram showing co-axial flow channels for fiber generation in microfluidic platforms. (**c**) Fiber shapes. Adapted from [12].

In the microfluidic fabrication of polymeric microfibers, the complete mixing of the different solutions is fundamental to obtaining homogeneous filaments [65]. Therefore, it is important to discuss some of the most common methods used to achieve mixing and how they can affect the geometry of microfluidic devices. These include the following:

(i). Parallel lamination (Y- or T-junctions, see Figure 1): classic mixers are based on merging two incoming streams without perturbation. As the two streams concurrently flow down the microchannel, the concentration gradient between the two laminar streams induces diffusional mixing. The incoming streams can be positioned in a converging configuration (Y-mixer) or in an opposing configuration (T-mixer) with respect to the main flow channel;
(ii). Dean vortices (Figure 2a): a serpentine channel, consisting of a series of opposite turns, can slightly improve mixing due to secondary flows in the direction perpendicular to the main flow.

Microfluidic systems are characterized by laminar flows both in the input channels and in the main channel, wherein the fluids contact [12]. When two or more different laminar streams come in contact with each other, they will not mix, except only through diffusion at the interface. In this way, a coaxial laminar flow is obtained through phase separation. Indeed, in most cases, a sample fluid and a sheath fluid are introduced into separate input ports and meet at a channel intersection: the sheath flow surrounds the sample flow, which is then solidified via cross-linking reactions, producing a fiber (Figure 2b).

Microfluidic spinning platforms have been used to produce different fiber shapes (Figure 2c), depending on the anatomical site where the fibrous scaffold has to be implanted:

- Solid/Porous cylindrical fibers: this is the most popular microfluidic spun-fiber shape. These fibers are comparatively easy to spin and can readily encapsulate cells without seriously damaging them;
- Tubular fibers: tubular fibers have been investigated for use in the engineering of neurons, muscle fibers and blood vessels;
- Hybrid fibers: these are microfibers with a heterogeneous distribution of materials and/or cells, as it has been shown that, in living organisms, the positioning of different materials and structures at the nano- and micro-scale facilitates the growth of organs or structures;
- Fibers with a more complex shape: specially designed microfluidic chips can be used to produce microfibers with size-tunable grooved microstructures. The number and dimensions of a few micrometer scale grooves can be controlled by changing the grooved patterns on slit-shaped channels.

### 4.3. Three-Dimensional Structures

A three-dimensional (3D) scaffold is a bioinspired structure that reproduces the natural ECM, on which the seeded cells attach and proliferate in order to regenerate damaged tissue. It must provide temporary mechanical support and proper physicochemical characteristics in order to promote cell adhesion, migration and organization.

In recent years, several methods have been developed for 3D scaffold fabrication, ranging from conventional methods, such as melt molding, freeze-drying and gas foaming, to advanced rapid prototyping techniques, such as 3D printing, fused deposition modeling and selective laser sintering. However, the porosity of these scaffolds is not adequate in terms of pore dimensions and pore interconnectivity. Moreover, these fabrication methods generally require additives and post-processing steps that may not be cytocompatible and can preclude cell encapsulation during scaffold fabrication.

An emerging paradigm in the production of porous scaffolds for tissue engineering is their assembly from microparticles or microfibers used as building blocks. This approach involves fabricating microparticles/microfibers in a preliminary step, packing them together, and then cross-linking them into a 3D structure. Due to the void spaces between the building blocks, this approach results in scaffolds with a highly interconnected microporous structure without requiring porogen or foaming agents.

When scaffolds are fabricated using building blocks, size uniformity is essential for obtaining a desired geometry with specific shapes and sizes that mimic functional tissue units [39]. In recent years, microfluidics has emerged as a valid option for the fabrication of microparticles and microfibers used as building blocks for 3D scaffolds.

Compared to the standard fabrication technologies of 3D scaffolds, microfluidics-based ones offer several advantages, such as the possibility of obtaining particles and fibers with uniform dimensions. Thus, a 3D scaffold with extremely precise geometry, pore distribution and uniform pore size can be obtained. Moreover, the pores are highly interconnected, which is a characteristic difficult to achieve using a standard fabrication method, for example, gas foaming. Fabricating a 3D scaffold using a microfluidic device also represents a cheaper manufacturing technique when compared to soft lithography or 3D printing, for example.

Since a microfluidic chip can also be precisely regulated to obtain a specific particle or fiber diameter, this method represents an innovation in tissue engineering. In fact, it offers the possibility of fabricating scaffolds with specific geometry and properties suitable for cell adhesion, migration and proliferation.

An overview of microfluidic-fabricated 3D scaffolds for tissue engineering obtained through the assembly of microparticles or microfibers as building blocks is provided in Table 3.

#### 3D Scaffold Fabrication by Microfluidic Devices

Microfluidic devices used to produce microparticles and microfibers as building blocks for 3D scaffolds are analogous to those already illustrated in Section 4.1 and Section 4.2, respectively. The overall procedure to obtain 3D scaffolds starting from particles/fibers is illustrated here.

The first step in the production of both porous and fibrous scaffolds is the preparation of a polymer solution that consists of a small amount of polymer (about 7 wt% gelatin [68,73], 1.5 wt% alginate [66] or 10 wt% GelMA [71] for microparticles and 2 to 3 wt% alginate for microfibers [13,74]) dissolved in a medium, such as Dulbecco’s Phosphate-Buffered Saline (DPBS) [14].

Starting from microparticles, both hollow particles and dense particles can be fabricated for use as building blocks. When fabricating hollow particles, a surfactant is added to the polymer solution at a concentration below 1 wt% in order to reduce surface tension and promote hollow microparticle formation. In contrast, to produce dense microparticles, a surfactant is generally added to the oil phase, which flows in the side channels [68]. If GelMA is used for particle production, a photo-initiator is added to the polymer solution in order to allow photo cross-linking [14].

Once the polymer solution (dispersed phase) is prepared, it is injected into one of the input channels at the same time as the continuous phase, which can be an oil/surfactant one, a sheath fluid or a gas. During this procedure, the device temperature can be kept around 40–45 °C to prevent the liquid-to-solid transition of the polymeric solution [14,68,73].

In order to obtain hollow particles in the output channel, gas pressure and polymer solution velocity must be controlled and set to a minimum of 0.34 atm and 20 µm min^−1^, respectively [66,67,68,73].

For dense particle production, in addition to the speed of the polymer solution, the oil flow must be properly set since bead diameter depends on it. Starting from a minimum of 10 µL min^−1^, depending on the polymer concentration, microparticles with a diameter of around 100 µm can be obtained. By increasing the oil flow and reducing the polymer solution speed, particles of smaller diameter (around 70 µm) can be obtained [15].

**Figure 3:**
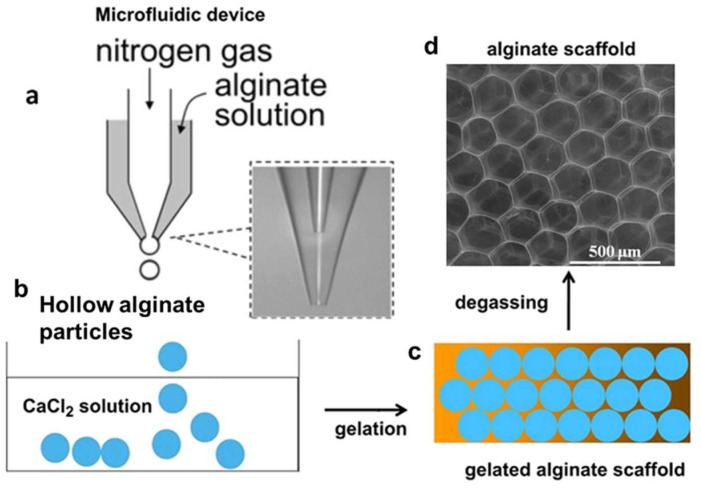
Procedure for the fabrication of highly organized 3D alginate scaffolds using a microfluidic device: (**a**) Production of hollow alginate microparticles using a two-channel fluid jacket microencapsulator; (**b**) gelation of alginate microparticles by ionic cross-linking with calcium ions; (**c**) obtainment of gelated alginate scaffold; (**d**) obtainment of alginate scaffolds with interconnected pores after treatment in a vacuum system overnight, to remove air bubbles. Adapted from [66] (**a**,**d**) and [67] (**b**,**c**).

Hollow microparticles are collected as a monodisperse foam and then cross-linked using different techniques depending on the polymer used. For example, alginate microparticles are generally cross-linked in a calcium chloride solution in order to obtain a gelled porous scaffold. Then, the scaffold undergoes degassing under a vacuum system overnight to remove air bubbles, allowing pore interconnectivity [64,66,67,73] (Figure 3). Gelatin microparticles generally undergo gelation immediately after being collected as a foam, simply reducing the working temperature.

**Figure 4:**
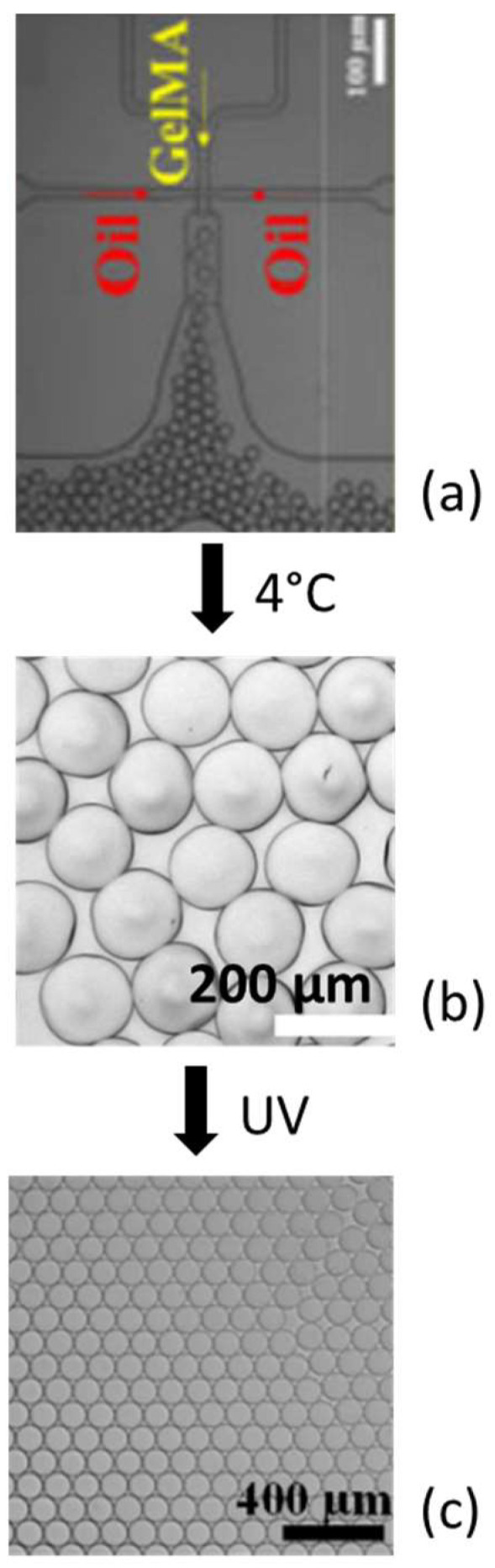
Procedure for the fabrication of 3D GelMA scaffold using GelMA microparticles as building blocks [14,15]. (**a**) GelMA microbeads production within a microfluidic device; (**b**) physically cross-linked microparticles obtained by cooling and excess oil removal; (**c**) microporous scaffold obtained by microparticles cross-linking through UV light exposure. Adapted from [14].

Considering the dense microparticles, which are generally made of GelMA, these are assembled to form a three-dimensional scaffold by annealing. The microparticle suspension in oil is collected at 4 °C overnight for physical cross-linking. Then, the excess oil is eliminated through pipetting, and the surfactant is removed after centrifugation. The concentrated microparticles suspension is later cross-linked through UV light exposure (Figure 4).

Considering microfibers, the microfluidic spinning technique is generally implemented through the use of a flow-focusing microfluidic device, using sodium alginate and calcium chloride as spinning and sheath fluid solutions, respectively. Alginate microfibres are formed in the output channel due to the rapid ion exchange at the intersection of the inlet channels within the chip [13,74]. The deployment of calcium chloride is crucial for the cross-linking phase that occurs in the output channel. Ca^2+^ ions diffuse into the spinning solution and interact with the highly negatively charged alginate chains, enabling fiber formation. The fibers can be fabricated into different shapes, depending on the arrangement of the two solutions in the microfluidic device. Other than solid microfibers, a microfluidic device can yield hollow microfibers, by injecting the polymer solution into the side channels and calcium chloride into the core channel. Additionally, cells can be encapsulated in microfibers, through the dispersion in one of the solutions injected in the microfluidic channels [64,72].

**Figure 5:**
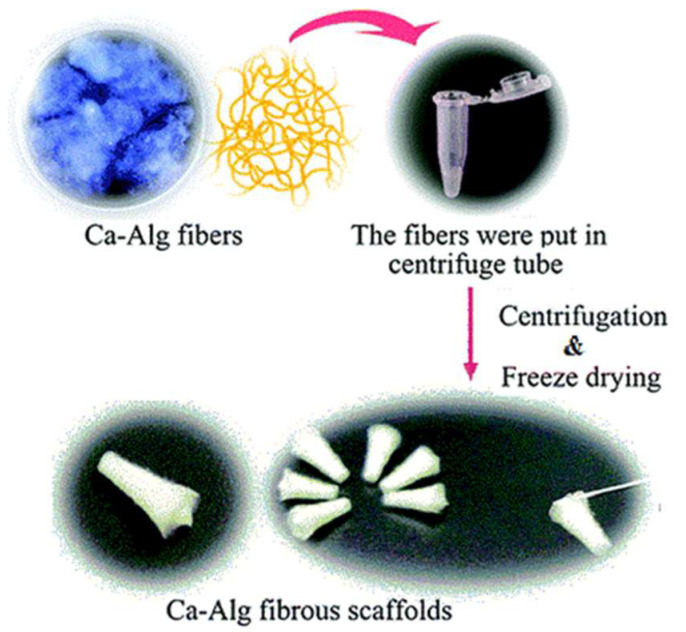
Fabrication procedure to obtain a fibrous scaffold from alginate fibers. Adapted from [13].

Once the fibers are produced, they are collected in a centrifuge tube, undergo centrifugation in order to discard the exceeding supernatant and then vacuum lyophilized in a freeze dryer in order to be packed before use (Figure 5). Otherwise, the fibers can be woven to obtain a 3D scaffold [64].

## 5. Tissue Engineering Applications

### 5.1. Bone and Cartilage

Bone and cartilage injuries occur due to various reasons, including degenerative, surgical and traumatic processes, significantly compromising quality of life. Currently, millions of patients are suffering from bone and cartilage defects, and the clinical need to effectively treat such conditions is expected to increase as aged populations continue to grow. Tissue engineering approaches emerged as a potential alternative therapeutic process to treat injured patients with minimally invasive techniques. The osteoblasts, chondrocytes and mesenchymal stem cells obtained from the hard and soft tissues of patients can be expanded in culture and seeded onto a scaffold, which will slowly degrade and resorb as the new tissue grows in vitro and/or in vivo. The use of a scaffold can overcome the limitations related to simple cell transplantation, in particular, retention and engraftment. A number of biodegradable and bioresorbable materials (including natural polymers, such as collagen and chitosan) and scaffold designs (including microparticles, microfibers and 3D porous systems) have been experimentally and/or clinically studied for the tissue engineering of bone and cartilage. Studies concerning scaffolds for orthopedic tissue engineering produced through the use of microfluidic devices are reported below.

Starting from microparticles, Zhao et al. developed injectable stem-cell-laden microparticles for bone regeneration [42]. Microparticles were produced using a GelMA solution as an aqueous phase and a perfluorinated oil as the oil phase in a microfluidic flow-focusing device. GelMa microparticles with a diameter of around 160 µm were obtained and cross-linked using UV radiation. The osteogenic potential of bone-marrow-derived mesenchymal stem cells embedded within GelMA microparticles was evaluated both in vitro and in vivo. The obtained results demonstrated that GelMA microparticles could maintain stem cell viability, support cell spreading and migration from the interior to the surface, enhance cell proliferation and increase mineralization.

Considering cartilage regeneration, alginate nanogels loaded with transforming growth factor beta 3 (TGF-β3) were produced using a T-junction microfluidic device in order to achieve a controlled release profile of TGF-β3 during the chondrogenic differentiation of mesenchymal stem cells [43]. Nanoparticles with a smaller size and a higher monodispersity, with respect to bulk synthesized nanogels, were obtained. Nanoparticle size was controlled by fine-tuning the ratio of the flow rates in the microfluidic device. As a consequence of the uniform size, TGF-β3 with sustained release and a reduced burst release were obtained. Furthermore, the ability of TGF-β3-loaded nanoparticles to promote the chondrogenic differentiation of mesenchymal stem cells has been demonstrated in vitro.

Regarding the use of microfibers for bone repair, different microfluidic approaches for their production have been developed.

**Figure 6:**
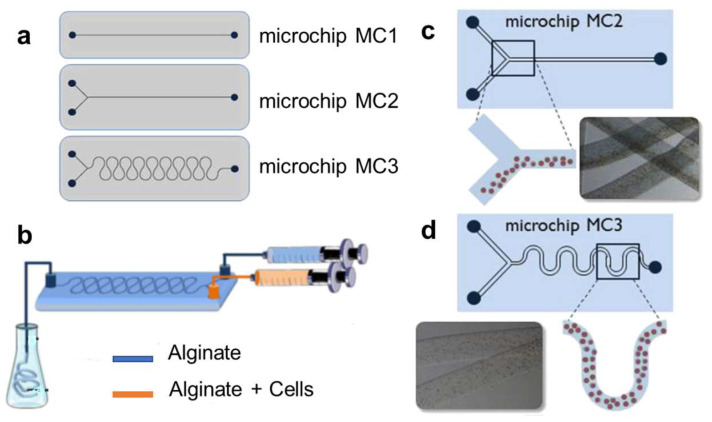
(**a**) Different microfluidic chips morphologies for the production of alginate microfibers; (**b**) microfluidic platform set-up; (**c**) representation of the cell segregation within MC2 microchip; (**d**) homogeneous cell distribution within MC3 microchip. Adapted from [53].

In a work published by Angelozzi et al., bioinspired microfibers based on alginate, eventually in combination with gelatin or urinary bladder matrix (UBM), were prepared using different microfluidic chips (Figure 6) [53].

In particular, the microchips included the following: a one-inlet straight channel microchip (MC1); a two-inlet straight channel microchip (MC2); and a two-inlet snake micromixing chip (MC3). Alginate microfibers were prepared by using microchip MC1. The procedure utilized a syringe–pump system, which leads the flow of sodium alginate solution through the microfluidic chip at a specific rate (that is adjusted to obtain the requested size of microfibers). The terminal part of the microchip outlet tube was perpendicularly immersed in a water-gelling solution containing barium chloride (BaCl_2_), calcium chloride (CaCl_2_) or strontium chloride (SrCl_2_): here, the polymer stream was gelled to produce the ionically cross-linked alginate microfibers. A more efficient approach for applications in bone tissue engineering is concerned with the production of composite alginate microfibers (containing SaOS-2 human osteosarcoma cells, ECM-derived components, or both), employing a two-inlet chip (MC2 or MC3). The use of a chip with two inlets allowed the production of microfibers containing a desired number of cells simply by adjusting the concentration of cells within the microfibers and the flow rates of the two independent pumps. A sodium alginate suspension containing SaOS-2 cells and a sodium alginate solution containing an ECM-derived component (i.e., gelatin or UBM) were delivered via the two inlets of the microchips MC2 or MC3. A water solution of BaCl_2_ was used as a gelling solution, producing the final alginate/gelatin and alginate/UBM microfibers. Optical stereo photomicrographs showed a segregated distribution of cells along the microfibers produced with the straight channels microchip MC2. The cells were only embedded along the side of the microfiber corresponding to the inlet position of injection. This was explained as a consequence of the fact that the dispersion of particulate matter under the laminar fluid flow conditions is scarcely affected by passive molecular diffusion. Moreover, the high viscosity of the polymeric solution and the use of particulate elements (such as cells), which are characterized by low diffusion coefficients, resulted in a poor dispersion of the particulate phase (segregation) within the microfiber structure. On the contrary, a snake-type micromixing channel geometry (microchip MC3) allowed the mixing of alginate and cells, producing a transverse transport inside the microfluidic device and promoting a homogeneous distribution of cells. In both approaches, the microfiber diameter was mainly affected by the pumping rate, the inner diameter of the outlet tube and the composition of the gelling solution; by varying these parameters, microfibers with diameters within the range from ~600 to ~2500 µm were obtained. Both in composite and plain microfibers, the abilities of the scaffold to support cell adhesion, viability, proliferation, 3D colonization and osteogenic differentiation were investigated, showing that the use of ECM-derived-components in combination with alginate produced microfibers with greater potential for bone-tissue engineering.

Microfluidic-fabricated microfibers have also been investigated for cartilage repair [54]. Cell-encapsulated microfibers were prepared by using chondrocytes isolated from patient nasal septum cartilage after a nasal cavity surgery. Chondrocytes were suspended in an aqueous solution of sodium alginate. The chondrocytes/alginate suspension and the CaCl_2_ solution were then flowed into a flow-focusing microfluidic device under the precise control of syringe pumps, converging at a T-junction. In the gelation region, the diffusion of calcium ions through the interface of the solution occurred, allowing microfiber formation. The gelled alginate microfiber was ejected from the outlet of the microchannel to the CaCl_2_ reservoir, in which the microfiber cross-linking was completed. The flow rate of each solution could be modulated to obtain microfibers (with a circular cross-section) with different diameters, ranging from 138 µm to 185 µm. The chondrogenesis and the increase in major markers of chondrogenesis, such as GAGs and collagen, were evaluated by both histological and biochemical analyses, and the obtained results showed the potential of cell-laden microfluidic fabricated alginate microfibers for cartilage repair.

Cartilage tissue regeneration has also been investigated both in vitro and in vivo on mice using 3D porous scaffolds fabricated via microfluidic technology, as conducted by Wang et al. [66,67]. A microfluidic device was used to fabricate a highly organized 3D scaffold based on hollow alginate droplets. The droplets were injected into a calcium chloride solution and gelated as empty microparticles by ionic bonding via Ca^2+^. The gelated alginate scaffold was then placed in a vacuum system overnight to remove air bubbles and create a system with interconnected pores. The scaffold was then seeded with chondrocytes or adipose-derived stem cells. The obtained results showed appropriate cell proliferation: the chondrocytes aggregated as spheroids without any transformation, allowing the generation of a cartilage-like matrix within a few weeks.

### 5.2. Heart

Heart diseases are a leading cause of death worldwide. The adult mammalian heart is among the least regenerative organs; thus, cardiomyocytes lack intrinsic regeneration capabilities. Currently available clinical treatments are not able to regenerate the injured heart, and, therefore, patients may experience heart failure. For this reason, the heart is one of the most important topics within tissue engineering research. Cardiac tissue engineering (CTE) aims to create contractile heart muscle tissue, leading to cardiac repair.

Scaffolds constitute a major component in various CTE strategies as stand-alone treatments or in combination with cells and/or bioactive molecules. CTE scaffolds aim to mimic the structural and architectural properties of the native cardiac ECM and enable controllable and efficient cell stimulation. Microscopic and submicroscopic features of scaffold structure have a very important influence on cardiac cell adhesion and growth.

**Figure 7:**
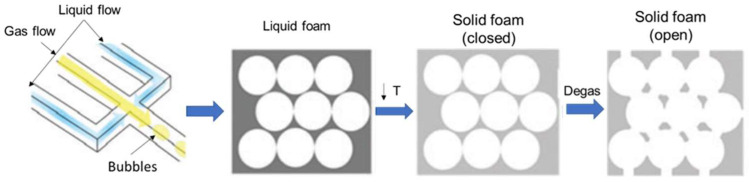
Flowchart of gelatin and gelatin/collagen 3D scaffold formation by assembling microbubbles as building blocks. A liquid foam made by hollow microparticles (bubbles) was collected from the microfluidic device; lowering temperature produced foam gelation; open solid foam (3D scaffold) obtained by degassing. Adapted from [68].

In addition to conventional scaffold fabrication techniques, myocardial tissue regeneration using microfluidic fabricated 3D scaffolds has been investigated. According to Mei et al., gelatin and gelatin/collagen porous three-dimensional scaffolds, produced using microfluidics, represent promising solutions for cardiac tissue engineering [68]. Three-dimensional scaffolds were produced using gelatin and gelatin/collagen microbubbles obtained through microfluidic channels. The liquid foam collected from the microfluidic device was turned into a solid foam by lowering the temperature, and then the solid foam was placed into a vacuum oven to obtain a 3D scaffold (Figure 7).

The 3D scaffolds produced offered a high mass transfer efficiency and variable pore sizes, which can be adapted to the various needs of different cell types. In addition, the fabrication procedure was effortless and fast. The mechanical characterization of the gelatin scaffolds was shown to have more suitable mechanical properties for cardiomyocyte culture with respect to the gelatin/collagen scaffold. Primary neonatal mice cardiomyocytes were successfully harvested and cultured on these scaffolds while maintaining their in vivo morphology. It was observed that if the cell density was high enough, the cells were able to connect with each other through pore interconnections, forming three-dimensional cardiomyocyte sheets. In addition, the scaffold was able to support cardiomyocyte spontaneous contraction up to 25 days after seeding.

Another example of a scaffold for CTE obtained through the use of a microfluidic device concerns the synthesis of GelMA microgels [44]. Aqueous GelMA droplets were produced through a microfluidic device with a flow-focusing channel geometry and subsequently photo-cross-linked to obtain microgels [44]. By changing the ratio between the flow rates of the aqueous phase and the oil phase, it was possible to control the size of the microgels. Cardiac cells were seeded and cultured on a microgel surface. Rapid cell adhesion, good proliferation and high viability (around 90%) were reported, suggesting their potential as an injectable scaffold for cardiac tissue engineering. In addition, it was demonstrated that after cell adhesion on the microgel surface, it was possible to coat them with biocompatible and biodegradable silica hydrogels in order to protect the cells from oxidative stress, which is often encountered during and after injection into host tissues.

### 5.3. Wound Healing

As the population ages, there is a growing clinical need for improved wound-healing therapies due to an increase in diabetes and associated skin wounds. In recent decades, there have been significant advancements in the manufacturing of wound-care products.

Tissue engineering approaches for wound healing and skin regeneration have been developed, and the delivery of cells, mainly mesenchymal stem cells, within scaffolds is one of the main strategies developed.

There are key advantages to the use of cell-seeded scaffolds over cell-only therapies in wound healing, especially for larger wounds. Three-dimensional scaffolds provide greater coverage, as well as help maintain the integrity of the tissue architecture during wound healing.

Additionally, in the field of tissue engineering for wound healing, natural polymers such as collagen, chitosan, fibrin, cellulose and alginate are widely used as scaffold materials, while among the scaffold manufacturing techniques, there are also microfluidic ones, which allow the production of both fibrous and porous systems. Some examples are given below.

**Figure 8:**
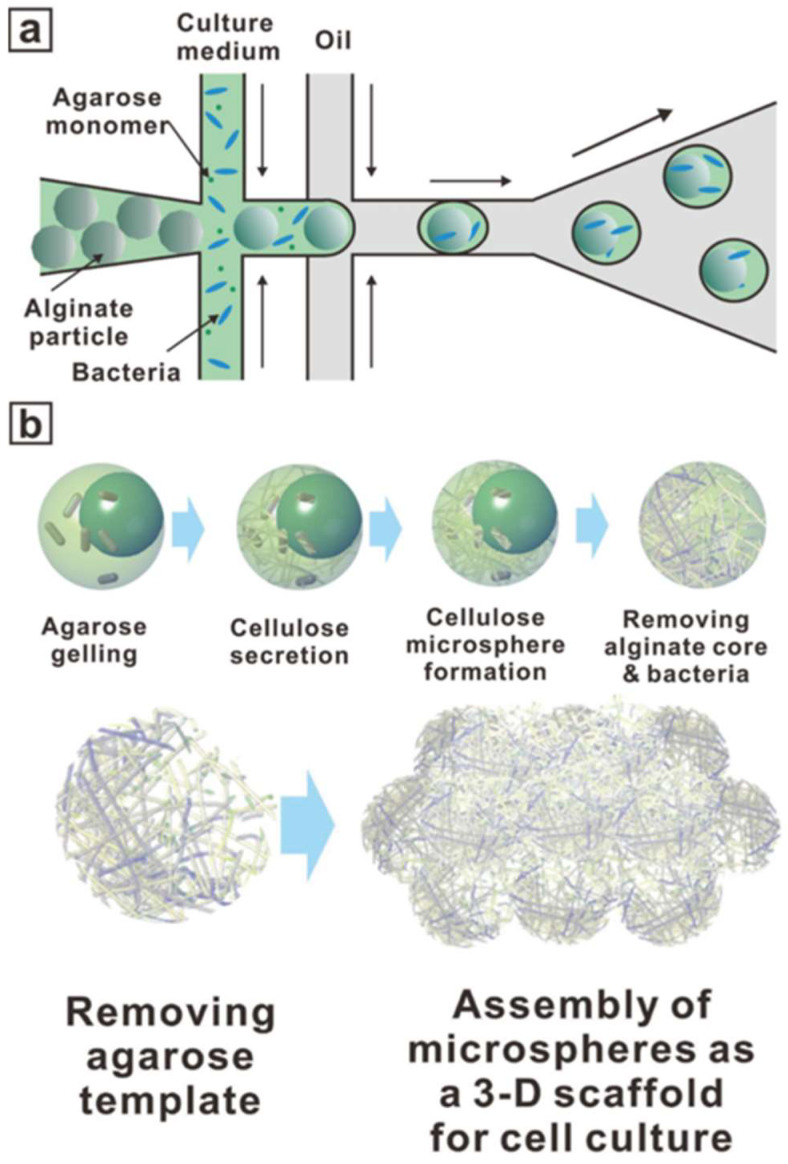
(**a**) Microfluidic device used for the production of double-layer alginate core agarose shell microdroplet; (**b**) following steps to produce hollow bacterial cellulose microsphere, including agarose gelling, cellulose secretion, removal of alginate core and bacteria, removal of agarose template and assembly of 3D scaffold, using hollow bacterial cellulose microspheres as building blocks. Adapted from [69].

Yu et al. developed a microfluidic process to produce hollow bacterial cellulose microspheres with desirable internal structure and morphology (Figure 8) [69].

Bacterial cellulose is a natural material with excellent mechanical properties and high water-holding capacity. It has been demonstrated as a promising candidate material for wound-healing applications in severely damaged skin and blood vessel replacement. As a consequence of its high chemical resistance, robust mechanical strength and insolubility, it is not easy to directly fabricate cellulose microspheres using microfluidics. Therefore, microfluidics was used to generate core–shell microparticles (with an alginate core and an agarose shell) to be used as a template for long-term static cultures of *Gluconacetobacter xylinus* (*G. xylinus*), a cellulose-producing strain. *G. xylinus* was encapsulated in the core of the microspheres and secreted cellulose fibers that became entangled within the shell and formed cellulose microspheres. Then, the hydrogel template was removed via thermal-chemical treatments, obtaining hollow cellulose microspheres. These hollow microspheres spontaneously assembled to form a robust scaffold with high porosity, which underwent a biological characterization in vitro and in vivo. *In vitro*, a high cell proliferation coupled with a good depth distribution were reported. *In vivo*, rapid wound healing in a Sprague Dawley rat skin model was promoted.

In another study, a fibrous scaffold was fabricated using microfluidic spinning and centrifugal reprocessing for the care of chronic wounds. Alginate was chosen for scaffold fabrication because its hydrophilicity provides high water absorption, ensuring wound moisturization. The results of the characterization tests performed on the fibrous scaffold showed a 3D structure similar to that of the ECM and excellent biocompatibility. Moreover, fibers loaded with silver nanoparticles exhibited broad-spectrum antibacterial activities, which could be beneficial for wound-dressing applications [13].

### 5.4. Liver, Pancreas and Kidney

The liver is the largest internal organ in the body and performs more than 500 different functions to maintain homeostasis in the body. Any liver damage may result in various metabolic and physiologic abnormalities, termed liver-associated pathologies. The liver possesses a unique capability for regeneration and the ability to fully restore its mass and function. However, in certain clinical conditions, the liver fails to regenerate itself and needs external assistance to resolve the complications. The strategies for treating pathological liver conditions mainly include orthotopic liver transplant (OLT) and the use of extracorporeal devices. OLT is still considered to be the gold-standard therapy for liver failure despite numerous problems, such as the scarcity of donors, limiting its use. On the other hand, extracorporeal liver support devices act as a temporary solution for patients awaiting donor organs or during the recovery phase of those who undergo organ transplantation. From this, it can be deduced that there is still a dearth of alternatives that could support the physiological function of the liver in a better way. In this regard, tissue engineering has emerged as a promising option. The first step in liver tissue engineering is the development of a scaffolding platform that could provide adequate biochemical, physicochemical, biomechanical and microarchitectural features that mimic native liver tissue. A wide variety of scaffolds have been reported in various forms, including hydrogels, films, cell sheets, etc. These constructs are often made using natural biomaterials such as chitosan, gelatin, alginate, collagen, etc. Studies involving the use of microfluidics to fabricate scaffolds for liver tissue engineering have also been conducted, and some of them are shown below.

**Figure 9:**
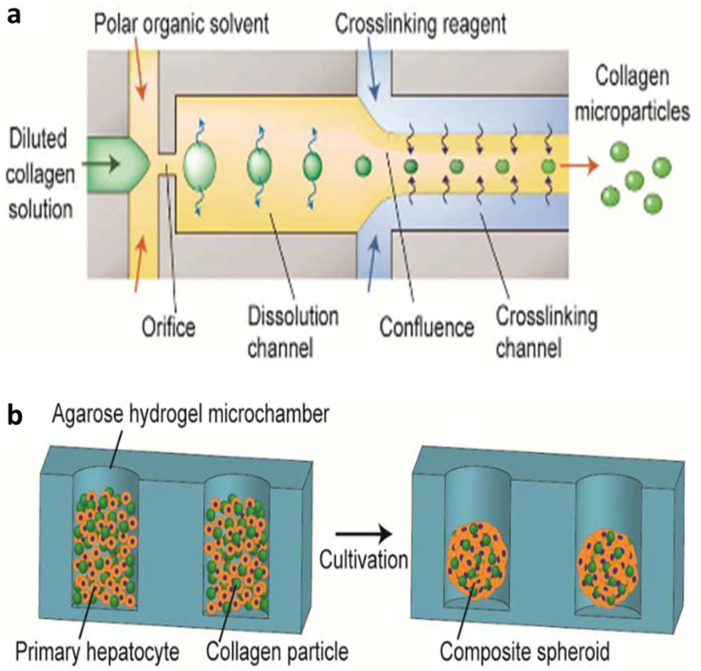
(**a**) Schematic illustration showing the microfluidic process for producing collagen microparticles; (**b**) formation of composite spheroids of primary rat hepatocytes, incorporating collagen particles. The formation of composite spheroids occurred in a non-cell-adhesive microchamber made of agarose hydrogel. Adapted from [37].

The first example concerns the development of collagen microparticles used to create composite spheroids of primary hepatocytes as structural units for the construction of the liver (Figure 9) [37].

Droplets of an aqueous solution of type I collagen were formed in a continuous phase of a polar organic solvent in a flow-focusing device. The obtained droplets were then cross-linked with glutaraldehyde to form disc-like particles with rough surfaces, which facilitate cell adhesion. Microparticle size could be regulated by regulating collagen concentration. Thanks to the cell adhesive properties of collagen microparticles, by introducing them with primary rat hepatocytes in an agarose hydrogel microchamber, composite spheroids were formed. The study showed that the ratio between collagen microparticles and cells should be 1:1 to preserve hepatocyte function, as demonstrated by the higher release of albumin and ornithine transcarbamylase (OTC) at this ratio. In addition, the particles produced using this method were highly condensed and solid and were, therefore, stable in 3D culture and showed stable cell adhesion on their surface. The use of collagen microparticles as particulate scaffolds for liver tissue engineering resulted in being advantageous because of their ability to preserve cellular functions, the simplicity of the operation and their high versatility.

Microfluidic fabricated fibers have also been investigated for liver tissue engineering.

**Figure 10:**
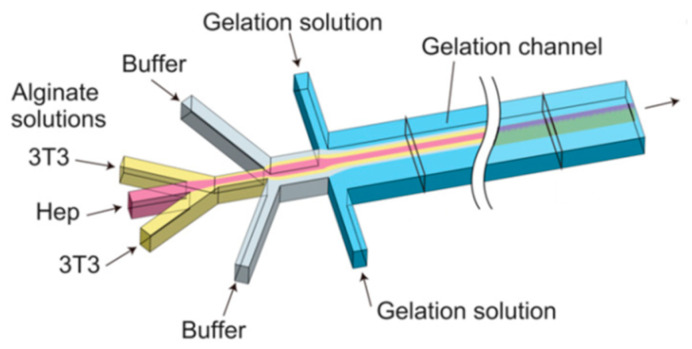
Microfluidic system for the production of sandwich-type alginate hydrogel microfibers that incorporate hepatocytes and 3T3 cells. Adapted from [56].

In a paper published in 2012 by Yamada et al. [56], a microfiber-based cell cultivation platform for the formation of hepatic micro-organoids was developed (Figure 10).

PDMS co-flowing microdevices were fabricated using standard soft lithography and replica molding techniques. The preparation of cell-laden alginate hydrogel fibers involved the injection of four different solutions simultaneously: a suspension of hepatocytes in a sodium alginate solution; a 3T3 cell suspension in a sodium alginate solution; an aqueous solution of BaCl_2_ (gelation solution); and a buffer solution. Cell-incorporating anisotropic Ba-alginate hydrogel microfibers were produced, in which hepatocytes at the center were closely sandwiched by 3T3 cells. Hydrogel fiber-based cultivation under high oxygen tension enabled the formation of heterotypic micro-organoids with a length of up to 1 mm and a diameter of 50 µm, mimicking the hepatic cord structures in the liver while maintaining a high hepatocyte viability. Moreover, long-term observation revealed a significant enhancement to hepatic functions because of cell–cell interactions, including albumin secretion and urea synthesis. These results demonstrated the potential of sandwich-type anisotropic alginate hydrogel microfibers for application in liver tissue engineering.

In another study, pure chitosan microfibers were generated using a coaxial microfluidic device [57]. The synthesis of microfibers occurred from the interface of two liquids: a chitosan solution as the core fluid and sodium triphosphate pentabasic (STP) as the sheath fluid. By adjusting the flow rates of the core and sheath fluids, fibers from 70 to 150 µm in diameter were generated, and they could be continuously extruded without disconnection. The extruded chitosan fibers were then wound onto a windowed polystyrene (PS) frame. To see if chitosan-based microfibers could be used to produce scaffolds for liver tissue engineering, HepG2 cells were cultured on them: it was observed that the cells were spontaneously self-aggregated, forming spheroid-like structures. The hepatic function of HepG2 cells on the chitosan microfibers was evaluated by measuring albumin secretion and urea synthesis over 5 days, showing high specific liver functions.

More recently, Liu et al. developed a microfluidic strategy to fabricate ultra-thin polyelectrolyte hollow microfibers to be used as cell carriers in the field of liver tissue engineering [55]. A PDMS microfluidic chip with four channels was fabricated using the rapid prototyping method. Solutions of methyl cellulose, chitosan, sodium alginate and calcium chloride were used as center flow, inner sample flow, outer sample flow and sheath flow, respectively. After methyl cellulose diffused out, calcium alginate hollow microfibers with a chitosan layer in the inner wall were first fabricated to ensure ionic interfacial interaction between alginate and chitosan. Then, the obtained microfibers were immersed in a PBS bath to remove the outer calcium alginate shell, thus producing ultra-thin polyelectrolyte hollow microfibers, with a diameter of 200 µm and a shell thickness of 1.3 µm. As liver tissue engineering requires a large number of cells assembled in a niche microenvironment, HepG2, as model cells, were encapsulated in the hollow structure of the microfibers. Cell morphology and hepatic functions were evaluated to explore the potential application of the developed microfibers in liver tissue engineering. The presence of the inner chitosan layer promoted cell adhesion, and the ultra-thin shell facilitated the exchange of nutrients and oxygen, thus promoting cell proliferation. HepG2 cells encapsulated in the microfibers maintained high viability, proliferation ability and hepatic functions during the 10 days of culture.

Considering the pancreas, it is well known that the loss of insulin-secreting ß-cells because of either autoimmune processes or surgical resection of the pancreas is responsible for Diabetes mellitus (DM). Although the current gold standard for the management of DM is exogenous insulin therapy in response to elevated blood glucose levels, this treatment option is inferior to continuous endogenous insulin secretion by ß-cells. Therefore, alternative therapies are needed that restore insulin-secreting function and avoid adverse effects, such as recurrent hypoglycemia and long-term complications. An alternative for patients refractory to exogenous insulin injection is islet transplantation. However, despite improvements in islet isolation and culture protocols and the investigation of different implantation sites for ß-cells, only 60%–85% of transplanted patients are insulin-independent 1 year after transplantation, and fewer than 20% of them remain insulin-independent for 5 years. In addition, an important limiting factor is the global shortage of suitable donor organs.

Pancreas tissue engineering approaches are therefore intended to overcome the current limitations in DM management. Cell-encapsulating hydrogels are currently used to host insulin-secreting cells (ISC). Scaffolds are intended to prevent anoikis, protect the patient from inflammatory and immunological host reactions and improve long-term viability.

However, the use of scaffold-based tissue engineering in the routine treatment of patients with DM is currently still hampered by several factors, including immunological host–graft reactions against cells and scaffolds. In this regard, a study is reported below in which immunoprotective microfibers were produced using microfluidics with the aim of avoiding a destructive host immune reaction against transplanted pancreatic islets [58]. Soft-lithography technology was used to create a microfluidic device with cylindrical and coaxial-flow channels integrated into a single platform. An alginate/collagen blend was chosen for fiber fabrication in order to mimic the native islet microenvironment. The collagen/alginate blend containing pancreatic islets isolated from rats was introduced in the central channel of the microfluidic platform, while a CaCl_2_ solution was introduced as a sheath fluid in the lateral microchannels. Two syringe–pumps controlled the flow speed of each fluid, which was modulated to obtain fibers with a uniform diameter of about 250 µm without protruding islets. Several in vitro and in vivo experiments were carried out to evaluate the feasibility of pancreatic islet entrapment in alginate/collagen fibers. The results were compared with free islets and islet encapsulation in pure alginate fibers. Pancreatic islets encapsulated in alginate/collagen fibers showed higher viability and normal insulin secretion. These results were attributed to the immunoprotection of the transplanted islets from host immune reactions and suggest that islet entrapment in newly designed alginate/collagen microfibers could be used for successful islet transplantation.

Another important organ, whose disease is a worldwide public health problem, is kidney. Current treatment methods are limited to life-long dialysis, which is capable of replacing renal filtration function by removing certain toxins from the blood, but it is unable to restore many other kidney functions, such as the production of erythropoietin and the activation of Vitamin D. This limitation of dialysis frequently leads to suboptimal quality of life and is further associated with high morbidity and issues of long-term survival.

Therefore, the only definitive treatment is kidney transplantation, but the scarcity of donor kidneys is perhaps the most important concern, as the supply of donor kidneys meets less than one-fifth of the demand. To address this issue, tissue engineering and regenerative medicine have been suggested as promising solutions to this problem through the development of tissue-engineered renal structures with normal renal functions. However, the regeneration of damaged renal structures, particularly the glomerulus, is very difficult. Therefore, the development of an in vitro glomerulus model is receiving significant interest to better understand this filtration unit.

In a recent study, microfluidics was applied to the production of a fibrous scaffold for a glomerulus-on-a-chip model. In vitro models should mimic the architecture and complexity of native organs. However, in currently available models, cells are cultured in a simplified 2D geometry without recapitulating the complex 3D structure of the kidney glomerulus. Xie et al. reported the development of a novel perfusable 3D engineered glomerulus based on a microfluidic extruded hydrogel scaffold [70]. Hollow microfibers, based on RGD-conjugated alginate, were generated from a coaxial microfluidic device. A novel chemically induced inflation method was then developed to generate a micro-convex topography on the hydrogel surface to mimic the micro-curved features of the looping capillaries. Endothelial cells were seeded in the perfusable lumen to generate a vessel-like perfusable tubular channel, while podocytes were cultivated on the surface of capillary loop-like structures to form a functional filtration barrier. The permeability of albumin from the vascular channel to the ultrafiltrate side was tested, demonstrating the successful fabrication of a 3D glomerulus filtration barrier.

### 5.5. Microvasculature

Blood vessels are a uniform part of the circulatory system whose function is to maintain a homeostatic environment in tissues by supplying oxygen and nutrients and removing metabolic byproducts. In particular, the microvasculature can be described as a system of small diameter vessels (usually less than 100 µm) that exhibit a high surface-area-to-volume ratio and enable the rapid exchange of fluids, solutes and cells across the endothelial layer between the vascular lumen and the surrounding tissue.

Vascularization is one of the most important challenges for creating in vitro large tissues. One strategy to promote tissue microvascularization is based on the inclusion of a prefabricated scaffold with a specific network geometry and seeded with ECs, into a separately fabricated scaffold, with the aim to act as a template for microvasculature network development.

Microfluidic-fabricated scaffolds based on natural polymers are currently also being explored for the development of functional and perfusable microvascular networks, which can be integrated into engineered tissues [75,76]. Engineered microvasculature has a two-fold objective: (i) facilitate functional tissue-regeneration post-implantation, and (ii) achieve more realistic in vitro models of natural tissues to be used for drug testing and for pathogenesis investigation.

Nguyen et al. investigated a microfluidic approach for the fabrication of cell-laden hollow fibers for endothelial barrier research [59]. A triple-flow PDMS microfluidic chip was designed and fabricated via replica molding. To ensure the fabrication of a perfect circular cross-section within the cylindrical microchannels, the ratio of radius and depth was fixed; this allowed the formation of mild and continuous coaxial flows for the fabrication of hollow fibers without employing complex glass microcapillaries. Three inlets in the device permitted the introduction of a sheath fluid (CaCl_2_), a sample fluid (sodium alginate) and a core fluid (mineral oil) whose flow was controlled using three separate syringe pumps. The cylindrical microfluidic device facilitated the movement of these fluids, according to the coaxial laminar effect, resulting in the formation of hollow fibers with a tubular shape. The hollow fibers were formed at the junction of the microdevice, where the sheath flow contacted the sample fluid, inducing alginate gelation by calcium ions. The mineral oil flowed coaxially without turbulence with alginate fluid, and it was responsible for the uniform space inside the solidifying alginate wall. The resulting hollow fibers had a 410 μm outer diameter and a 200 μm inner diameter. Various combinations of alginate and CaCl_2_ concentrations were evaluated to find the best one in terms of mechanical strength. The obtained hollow fibers also displayed flexibility, high permeability, biocompatibility and high adhesion and proliferation of human umbilical vein endothelial cells (HUVECs). Therefore, fully covered HUVEC fibers were further integrated into a neurovascular system and co-cultured with astrocytes to form an on-chip blood–brain barrier model for drug testing.

### 5.6. Nerve Tissue Engineering

Injuries to the central nervous system, which involve the disruption of axonal bundles, have a major impact on the population, healthcare, and the socio-economical field. After damage, central nervous system axons have an intrinsically limited capability to regenerate. Moreover, the loss of neuronal populations and synaptic connections is mostly irreversible due to the limited outgrowth capacity of mature neurons.

Conventional medicine does not have effective and successful treatments for these injuries, and the treatment of symptoms is often the only available solution. Nerve tissue engineering is currently looking to the use of biocompatible three-dimensional scaffolds in combination with cells and bioactive molecules in order to restore the integrity of the damaged tissue, respecting the original anatomy as much as possible while recovering its functionality. Different fabrication techniques, including microfluidics, are available to produce scaffolds that guide axonal outgrowth, such as aligned scaffolds, fibers or filaments and tubular structures. Some examples of 3D nervous tissue regeneration systems produced by microfluidic techniques are shown below.

Microparticles composed of collagen and alginate, produced using a customized microfluidic device, have been investigated for neural stem cell encapsulation because they combine the mild cross-linking conditions of alginate with the ability of collagen to promote cell adhesion [45]. Neural stem cells were suspended in a polymer solution containing type I collagen, alginate and microcrystals of CaCO_3_. The cell-laden polymeric solution was then extruded through the middle inlet, while the continuous phase and acetic acid were extruded through the other inlets to form microparticles. Neural stem cells encapsulated in collagen–alginate microparticles maintained cell viability for up to 21 days and retained multipotency as well as neuronal differentiation upon exposure to specific differentiation-inducing signals. After transplantation into an organotypic spinal cord injury model, the cell-laden composite microparticles exhibited effective retention of transplanted stem cells with a high survival rate over 10 days and their neural lineage-specific commitments.

**Figure 11:**
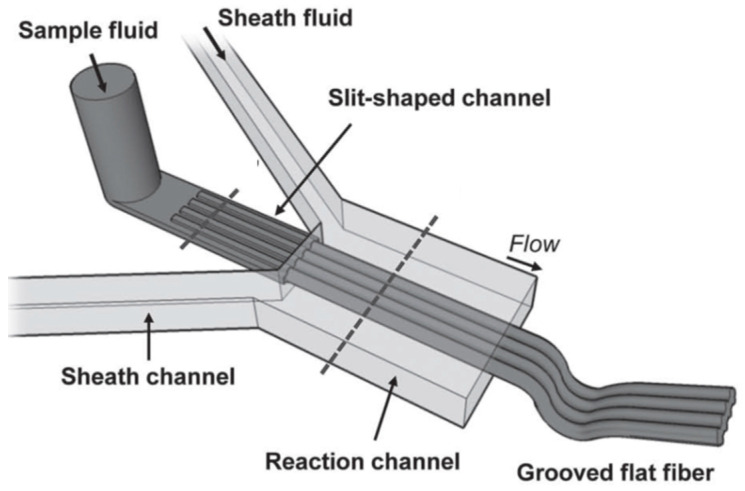
Microfluidic device for the production of flat fibers with microgrooves. Adapted from [60].

Kang et al. investigated the application of microfluidic spinning for the fabrication of flat alginate microfibers with size-tunable grooves, with the aim of promoting cell alignment and tested them with neuron cells (Figure 11) [60].

The microfluidic device for the production of grooved flat fibers consisted of a co-axial grooved slit channel constructed by aligning and bonding two grooved PDMS channels (fabricated using standard soft lithography methods) using an oxygen plasma treatment. The number and size of grooves were modulated by changing the groove pattern on the sample channel. Smooth flat fibers were produced using a non-grooved slit channel as a control. The sample fluid (sodium alginate) and the sheath fluid (deionized water solution of CaCl_2_) were introduced to the inlets of the device; the flow rate of each fluid was controlled using a syringe pump. Flat fibers were extruded in a Petri dish containing CaCl_2_, in which they were fully cross-linked. Then, the extruded fibers were wound around a rotating spool. To study the potential of the grooved and smooth flat microfibers, rat embryonic cortical neurons were isolated and then cultured on the fibers, evaluating cell morphology and alignment. On the smooth flat fibers, neuron cells accumulated at the edges and formed a random network of neurite connections across the fiber surface. On the contrary, in the case of grooved flat microfibers, neuron cells migrated along their groove ridges, showing an alignment of neurites along the longitudinal axis of the fibers. These results indicated that neurons are affected by the microtopographic features of the fibers, which guide cell migration, interaction, organization, and neurite orientation. The aligned neuron cell cultures suggest a potential application of grooved fibers as neuronal scaffolds that guide cellular morphology.

Recently, a flow-focusing microfluidic device was used to encapsulate adult rat hippocampal progenitor cells (AHPCs) within alginate-based fibrous hydrogels [61]. A solution of alginate mixed with a cell suspension was introduced, with a constant flow, into the core channel of the microfluidic device. A solution of calcium chloride and PEG was used as sheath fluid and pumped into the two side channels to help guide and solidify the core solution. Fibers exiting from the microfluidic device were introduced into a calcium chloride collection bath to complete the cross-linking. Then, cells encapsulated in microfibers were cultured for 4 days in vitro and recovered, to investigate the effects of the 3D microenvironment on stem cell fate. Post recovery, the cells were cultured for an additional 24 or 72 h in vitro before being fixed. The obtained results showed that encapsulation within alginate microfibers promotes cell proliferation and supports neuronal and glial differentiation.

### 5.7. Drug-Loaded Scaffolds

Tissue engineering scaffolds are used to create a suitable microenvironment for the recruitment, adhesion, proliferation and differentiation of cells. These processes can be further stimulated by the addition of bioactive agents, such as growth factors or drugs, which have an influence on cellular functions and tissue regeneration.

Controlled drug delivery can be accomplished by physically or chemically adsorbing the drug onto the surface of the scaffold, encapsulating the drug directly within the scaffold, or incorporating a drug delivery system into the scaffold. The subsequent release of the drug occurs by diffusion or due to degradation of the scaffold or encapsulating material. Drug release kinetic and duration can be controlled by altering several parameters, such as the dose of the drug added, the composition of the scaffold and of the delivery system, and the method of drug loading. Drug release kinetics and duration can be further tuned by coating the scaffold with substances, such as heparin, that specifically or non-specifically interact with the drug.

Porous scaffolds, as well as fibrous ones, can be loaded with bioactive molecules and then implanted in the patient’s body, where the molecules are released, allowing a better in situ regeneration of the damaged tissue that has to be treated.

Microfluidic fabricated scaffolds are also under investigation as platforms for drug delivery during tissue repair.

For example, Cai et al. developed a new drug-loaded porous scaffold based on a microfluidic droplet template for the prevention of intrauterine adhesions [71]. A capillary microfluidic device, assembled by coaxially aligning two (inner and outer) tubular capillaries inside a square capillary, was used for droplet production. The outer phase was an aqueous solution containing GelMA with a photoinitiator, sodium–alginate and an aqueous surfactant solution; a silicon oil was employed as the inner phase. The droplets were collected in a PDMS mold replicating the uterine cavity morphology of rats for subsequent in vivo tests. The droplets were solidified by UV irradiation and immersion in a calcium chloride solution. The obtained scaffold was characterized by a porous structure, good mechanical properties and flexibility, which is an important requirement for its implantation. In addition, it was possible to load and control the release of drugs to repair the damaged region. In vitro and in vivo biological characterization showed that the developed scaffold allowed better neovascularization, cell proliferation and endometrial repair, which prevent intrauterine adhesion due to drug release, gradually promoting the repair of the damaged tissue while cells proliferate.

### 5.8. General Tissue Engineering

In recent years, increasing progress in microfluidics is affirming its use in a wide range of applications, including tissue engineering. The results of several studies demonstrate the efficacy of combining microfluidics with tissue engineering. As already pointed out in the previous paragraphs, the use of microfluidic devices facilitates the creation of microfibers and microparticles that can be readily loaded with cells or therapeutic agents and whose properties are better controllable with respect to those of scaffolds produced by using traditional techniques. In addition to specific tissue engineering applications, several papers in the literature propose microfluidic fabricated scaffolds for tissue engineering applications in general. An overview will be provided in this section.

**Figure 12:**
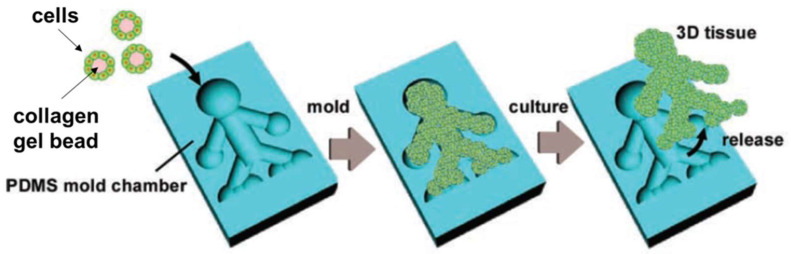
Formation of millimeter-scale 3D tissue architectures by molding monodisperse cell beads: cell beads are prepared by culturing cells over the surface of collagen gel beads; cell beads are stacked into the designed silicone mold to form 3D tissues; during culture, cells migrate and grow within the collagen gel beads and finally form the 3D tissues, that are released from the mold. Adapted from [51].

The first example concerns monodisperse microparticles, consisting of type I collagen, which were explored for the rapid bottom-up construction of millimeter-thick macroscopic tissues with complex microstructures (Figure 12) [51].

Collagen droplets were produced using corn oil with lecithin as the continuous phase in an axisymmetric flow-focusing device. Subsequent droplet gelation at 37 °C for 45 min led to the creation of collagen microparticles. The microparticles were then seeded with different cell types, such as NIH-3T3 fibroblast, HepG2 hepatocytes, human umbilical endothelial cells (HUVECs), primary neurons, primary rat hepatocytes and MIN6m9 pancreatic β cells. Collagen microparticles were then molded into a silicon chamber to assemble uniform and arbitrarily shaped macroscopic 3D tissue structures. In the mold, microparticles adhered to each other thanks to cell–cell connections. Moreover, the migration and growth of cells into the microparticles led to the contraction and decomposition of collagen, and subsequently, cell-dense tissue constructs could be released from the mold. As outlined by the authors [51], it was also possible to extend the lifetime of the tissue by increasing the diameter of the collagen microparticles. Therefore, using this approach, it is possible to obtain a macroscopic cell-dense tissue with a tunable lifetime.

In another work, Chau et al. reported the microfluidic fabrication of small monodisperse composite agarose/GelPh microparticles to be used for cell encapsulation [46]. The blend of agarose-GelPh was chosen to mimic the complex mixture of proteins and proteoglycans in native ECM. A microfluidic flow-focusing device was used. The channels were treated before use to make them hydrophobic. Aqueous solutions of agarose, GelPh and cross-linker were injected into the device as constituents of the droplet phase. A fluorinated oil containing a tri-block copolymer as a surfactant was used as the continuous phase. The microfluidic emulsification was carried out at 37 °C. The obtained microparticles first underwent agarose gelling under reduced temperature, and then GelPh enzymatically catalyzed gelation in the presence of a subtoxic concentration of hydrogen peroxide. Microparticle microstructure, morphology and stiffness were controlled in a throughput manner by changing the composition of the microparticles, with the final aim of developing a platform to investigate the role of the local microenvironment in cell fate.

**Figure 13:**
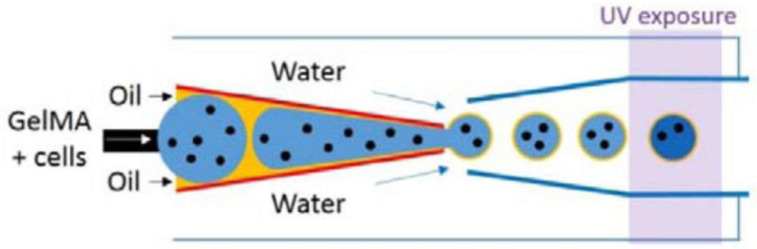
Schematic illustration showing a glass capillary microfluidic device used for the preparation of double emulsion drops with encapsulated cells. The innermost drops, based on a photo-cross-linkable polymer, are solidified to form microgels by UV exposure. Adapted from [50].

Cell-laden GelMA microspheres were fabricated by Choi et al. by using double-emulsion droplets with ultrathin oil shells as sacrificial templates for widespread applications in tissue engineering and cell therapies (Figure 13) [50].

Using a co-flow microfluidic device, a coaxial flow of an aqueous prepolymer solution, surrounded by an oil phase, was generated, resulting in monodisperse double-emulsion drops. The thin oil shell, in the double emulsion, spontaneously dewets upon the photo-crosslinking of the innermost precursor drop and subsequently transfer into an aqueous solution, resulting in the direct dispersion of microgels in an aqueous phase. This allowed biocompatible cell encapsulation by avoiding potential cytotoxicity from the long-term exposure of the cells to oil and surfactants. Madin–Darby Canine Kidney epithelial (MDCK) cells and NIH-3T3 fibroblasts encapsulated in these GelMA microparticles exhibited high viability and proliferation. However, while epithelial MDCK cells were characterized by a densely packed cellular organization, NIH-3T3 fibroblasts came out from the internal space of the microparticles and attached to their surface, bridging and aggregating neighboring microparticles. Interestingly, GelMA microparticles severely deformed from the original spherical shape and considerably reduced in size, most likely as a consequence of the compressive forces exerted by cell adhesion on hydrogel matrices.

Another example of microparticles prepared by microfluidics is the use of GelPh to encapsulate cells with the aim of creating artificial tissue constructs [49]. Sakai et al. developed an easy and useful method to encapsulate HepG2 cells in GelPh microparticles with a hollow core of less than 200 µm in diameter [49]. Cell-laden microparticles were first fabricated using a microfluidic flow-focusing droplet production system, which generated droplets of unmodified gelatin aqueous solution in a water-immiscible liquid paraffin flow. These gelatin microparticles were then used as templates for the production of hollow microparticles: using the same microfluidic system, they were coated with GelPh and then enzymatically cross-linked to obtain cross-linked GelPh microparticles (ECGelPh). Subsequent incubation at 37 °C produced the liquefaction of the enclosed microparticles based on unmodified gelatin. Therefore, enclosed cells grew in the hollow cores of the obtained microcapsules. Furthermore, cell-enclosing ECGelPh microcapsules were successfully coated with an additional adherent L929 cell layer. The enclosed cells were individually distributed immediately after encapsulation. After 24 h incubation, the formation of aggregates in the hollow cores was observed. The aggregates grew and almost occupied the hollow core after 4 days. Furthermore, 24 h after the seeding of the L929 cells on the surface of the microcapsules, these were connected by the adherent cells. These results showed the suitability of ECGelPh microcapsules for engineering tissue constructs composed of spherical aggregates surrounded and connected by a heterogenous cell layer.

Several reports on microfluidic fibers for general tissue engineering applications are also available in the literature.

Kim et al. developed microfluidic pH- and temperature-responsive hydrogel microfibers and microtubes for potential application in tissue engineering [62]. Multistimuli-responsive hydrogels in fibrous or tubular forms were prepared with a microfluidic device through photopolymerization using an alginate templating method. The microfluidic device used in this work consisted of two glass capillaries, with different sizes, for the production of microfibers and microtubes with controlled dimensions. The surfaces of the capillaries were made hydrophobic by immersion in a solution of octadecyltrichlorosilane/toluene, and they were then coaxially aligned, fixed and connected to syringe pumps. The first solution, containing monomers (N-isopropylacrylamide (NIPAAm) and sodium acrylate (SA) or allyl amine (AA)), alginate, a cross-linker and a photoinitiator, and the second, solution containing calcium chloride, were injected at constant and controlled flow rates through different capillary inlets. For the preparation of the microfibers, the monomer and calcium chloride solutions were injected into the inner and outer capillaries, respectively, and vice versa for the preparation of microtubes. When these solutions came into contact during coaxial flow, Ca^2+^ ions diffused into the monomer solution and formed tubular alginate templates. When the alginate-templated microfibers or microtubes emerged from the capillary, they were exposed to 365 nm UV radiation for in situ photopolymerization and then immersed in an ethylenediaminetetraacetic acid (EDTA) solution to remove the alginate template. This method produced cross-linked hydrogel microfibers and microtubes that exhibited fully reversible and repeatable volume change in response to temperature and pH changes. Indeed, repulsive forces between the ionized SA or AA groups caused by protonation/deprotonation of the acrylate or amine groups, respectively, lead to changes in the diameters and wall thicknesses of the fibers and tubes depending on the pH of the aqueous medium. These pH-dependent diameter changes were also linearly proportional to the SA or AA content of the copolymer microfibers. Microfibers also exhibited thermally responsive behaviors close to the lower critical solution temperature, thanks to the presence of poly(NIPAAm), which is a well-known thermally responsive polymer. To demonstrate the potential of these pH and temperature responsive microfibers for application in tissue engineering, HepG2 cell attachment was evaluated, and the results showed good cell adhesion.

**Figure 14:**
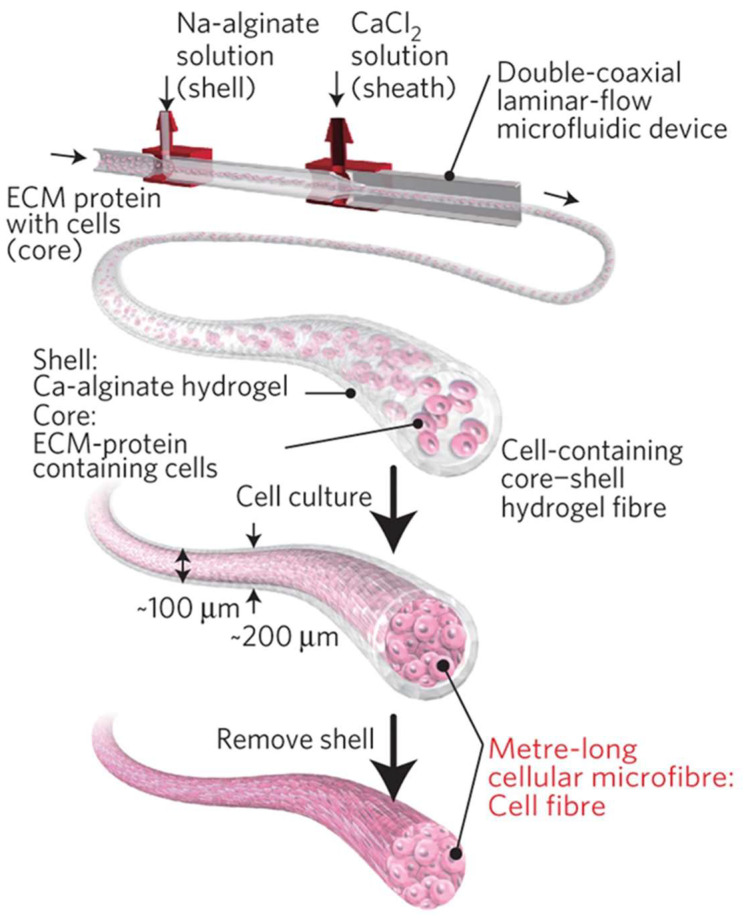
Overall approach to fabricate microfibers for the reconstruction of fiber-shaped functional tissues (such as nerve networks, muscle fibers and blood vessels), using a double-coaxial laminar flow microfluidic device. Adapted from [63].

In another paper, Onoe et al. developed a new microfluidic approach to create microfibers for the reconstruction of fiber-shaped functional tissues that mimic nerve networks, muscle fibers and blood vessels (Figure 14) [63].

The end product was a microfiber based on natural ECM proteins (pepsin-solubilized type-I collagen, acid-solubilized type-I collagen and fibrin) containing cells. A double-coaxial laminar flow microfluidic device, composed of glass capillaries and connectors, was used because ECM proteins need a longer gelation time than other hydrogels. The method for cell fiber production comprised three main steps. First, a core–shell hydrogel microfiber was formed using the microfluidic device. The core of the microfiber consisted of pre-gel-state ECM proteins containing cells, and it was encapsulated by a shell made of a Ca-alginate hydrogel. ECM proteins did not diffuse away from the core until their gelation, thanks to the outer shell, because the molecular weight cut-off of the Ca-alginate hydrogel was smaller than that of the ECM proteins. Thus, the ECM proteins remained as a microfiber within the Ca-alginate tubular shell and had sufficient time to turn into a gel. The second step was culturing the cells in this core to form a fiber-shaped cellular construct. The gelated ECM proteins in the core regulated cell migration and proliferation inside the tubular Ca-alginate shell, directing their essential morphological organizations and physiological functions. In the third step, the Ca-alginate shell was selectively digested by alginate lyase and removed to complete the creation of fibers that consisted only of cells and ECM proteins. The results of in vitro cell culture tests performed by using ten different types of cells showed that the microfibers produced with this method might find use as templates for the reconstruction of fiber-shaped functional tissues that mimic muscle fibers, blood vessels or nerve networks in vivo.

In a recent paper, Wang et al. developed a facile two-flow microfluidic system through which cell-laden hydrogel microfibers with various structures could be easily prepared in one step without changing the device [64]. A blend of alginate and GelMA was used as a scaffold material with the aim of improving the cell adhesion of alginate microfibers thanks to GelMA incorporation. Aiming to meet different tissue engineering needs, several types of microfibers with different structures, including single-layer, double-layer and hollow microfibers, have been prepared by merely changing the inner and outer fluids. Single-layer microfibers were subsequently seeded with mouse embryonic osteoblast precursor cells (MC3T3-E1). Cell-laden double-layer and hollow microfibers were prepared by directly encapsulating MC3T3-E1 cells or human umbilical vein endothelial cells (HUVECs) in the cores of microfibers during fabrication. The single-layer microfibers supported cell adhesion and growth on their surface, while double-layer and hollow microfibers supported cell proliferation within microfibers. The hollow microfibers also show good perfusion performance. Overall, the obtained results suggested potential applications of the produced microfibers in tissue engineering.

Considering 3D scaffolds, Lin et al. developed an efficient method to fabricate monodisperse foam scaffolds made of gelatin for 3D cell culture [73]. A planar flow-focusing microfluidic device made of PDMS was used to generate gelatin monodisperse bubbles that self-assemble into highly ordered flowing lattices collected into disc-shape reservoirs by using a solution of gelatin and Pluronic F127 in the liquid inlets and nitrogen in the air inlet. The liquid foam was gelled by decreasing the temperature to 4 °C to obtain a solid foam with closed pores made by bubbles. Finally, the closed-pore solid foam was transformed into an open-pore solid foam by degassing under a vacuum while immersed in a liquid cross-linking solution. Three different cell types were then cultured on the developed scaffolds. The cells displayed appropriate morphological and physiological characteristics: epithelial cells formed cyst-like structures and were polarized inside pores, myoblasts adopted a tubular structure and were fused into myotubes, and fibroblasts exhibited a wide variety of morphologies. These results demonstrated that scaffolds with uniform pores could thus provide a platform for the systematic study of 3D cell–matrix interactions.

In another study, Costantini et al. investigated the potential of microfluidic foaming within a flow-focusing geometry to produce 3D regular sponge-like polymeric matrices with tailored morphological and permeability properties for applications in tissue engineering [77]. Monodisperse alginate bubbles were produced within the flow-focusing device when the gaseous phase and the liquid phase (alginate solution) met orthogonally upstream of a tight junction: the gaseous phase penetrated inside the orifice, inflated a bubble, and was then squeezed by the liquid phase. When an acceptable amount of foam was collected, it was frozen in liquid nitrogen, lyophilized and then cross-linked. The authors demonstrated that it was possible to control pore size, pore volume, and the size of the interconnections among the pores, by simply changing polymer concentration, surfactant concentration, applied gas pressure and liquid flow rate. Overall, the obtained results demonstrated that the microfluidic-assisted synthesis of porous materials is a facile and versatile tool for tailoring the morphological and permeability properties of porous scaffolds.

More recently, Sheikhi et al. developed a novel facile, universal strategy to convert a thermosensitive material with cross-linkable moieties (GelMA) into a bead-based scaffold in order to overcome the current inability to combine pore interconnectivity and stiffness in injectable bulk hydrogels [14,15]. A PDMS flow-focusing device was used to produce a water-in-oil emulsion containing GelMA and a photoinitiator in the aqueous phase and a mineral oil with surfactant in the oil phase. GelMA beads with various sizes were produced by changing the GelMA solution concentration and the ratio of oil to aqueous flow rates. A suspension of surfactant-stabilized micron-sized beads in oil was obtained and stored at 4 °C overnight to create physically cross-linked GelMA beads. The microbeads were purified by excess oil using a secondary surfactant and, finally, chemically cross-linked using UV light exposure to create microporous scaffolds with high mechanical resilience. The obtained scaffolds were able to promote cell adhesion, proliferation and rapid 3D seeding at a high polymer concentration, which would otherwise be impossible for bulk GelMA.

## 6. Conclusions and Future Outlook

As shown in this review, microfluidic methods offer great advantages in the field of tissue engineering, allowing the fabrication of scaffolds that better mimic the ECM and offer a better environment for cells with respect to the matrices produced using traditional techniques. The advantages, offered by using microfluidic fabrication techniques over traditional ones, are pore interconnectivity, tailored pore dimensions for a specific kind of cells, homogeneous pore distributions and dimensions and higher porosity, which allow a better flow of nutrients to cells and lower manufacturing costs.

Since scaffolds fabricated using microfluidics offer many advantages, their application is rapidly spreading to the regeneration of different tissues, including cartilage, myocardium, skin, blood vessels, the kidneys and the liver. More recently, microfluidic fabricated three-dimensional scaffolds have also been investigated for applications in cellular and gene therapies, as well as for the development of in vitro three-dimensional tissues and organ models, with promising results.

In view of a possible clinical application of microfluidic fabricated scaffolds, further research is necessary, in particular with reference to the rate of production. The parallelization of identical microfluidic devices to scale up the production should be further developed to produce scaffolds combining large dimensions with complex geometries. Moreover, microfluidic scaffolds obtained from natural polymers often have poor mechanical properties and, therefore, improving this aspect will be necessary for their application in the treatment of both soft and hard tissues.

Once these improvements are achieved, microfluidic fabricated scaffolds could significantly contribute to the development of engineered tissues and organs.

## Figures and Tables

**Table 1:** Microfluidic fabrication of microparticles used as scaffolds for tissue engineering applications.

Biomaterial	Microfluidic Design	Cross-Linking Strategy	Tissue Engineering Application	Ref.
GelMA	Flow-focusing	Photo	Bone	[42]
Alginate	Flow-focusing (T junction)	Ionic (in situ)	Cartilage	[43]
GelMA + silica	Flow-focusing (cross junction)	Photo	Heart	[44]
Collagen	Flow-focusing	Chemical	Liver	[37]
Alginate/Collagen	Customized	Ionic	Nerve	[45]
GelPh/agarose	Flow-focusing	Physical + enzymatic	General	[46]
Alginate	Centrifugal	Ionic	General	[47]
GelMA	Double flow-focusing	Photo	General	[48]
GelPh	Flow-focusing	Enzymatic	General	[49]
GelMA	Co-flow	Photo	General	[50]
Collagen	Flow-focusing	Physical	General	[51]

**Table 2:** Microfluidic fabrication of microfibers used as scaffolds for tissue engineering applications.

Biomaterial	Microfluidic Design	Cross-Linking Strategy	Tissue Engineering Application	Ref.
Alginate, Alginate/gelatin, Alginate/urinary bladder matrix	One-inlet straight channel microchip, two-inlet straight channel microchip, two-inlet shake micromixing chip	Ionic	Bone	[53]
Alginate	Flow-focusing (T junction)	Ionic	Cartilage	[54]
Alginate and chitosan	Four channels chip	Ionic	Liver	[55]
Alginate	Flow-focusing	Ionic	Liver	[56]
Chitosan	Co-axial		Liver	[57]
Collagen/alginate	Co-axial	Ionic	Pancreas	[58]
Alginate	Co-axial	Ionic	Microvasculature	[59]
Alginate	Co-axial	Ionic	Nerve	[60]
Alginate	Flow-focusing	Ionic	Nerve	[61]
Alginate, PNIPAAM-co-sodium acrylate, PNIPAAm-co-allyl amine	Co-axial flow	Ionic + Photopolymerization	General	[62]
Collagen, fibrin	Double co-axial flow	Physical	General	[63]
Alginate/GelMA	Two-flow	Ionic + Photochemical	General	[64]

**Table 3:** Microfluidic fabrication of 3D scaffolds for tissue engineering, using microparticles and microfibers as building blocks.

Biomaterial	Microfluidic Design	Cross-Linking Strategy	Tissue Engineering Application	Ref.
Alginate	Two-channel fluid jacket microencapsulator bubble formation	Ionic	Cartilage	[66,67]
Gelatin, Collagen/gelatin	Flow-focusing	Chemical	Heart	[68]
Bacterial cellulose	Flow-focusing		Wound healing	[69]
Alginate	Flow-focusing	Ionic	Wound healing	[13]
RGD-conjugated alginate	Co-axial	Ionic	Kidney	[70]
Alginate, GelMA	Co-axial	Photo + ionic	Endometrium repair	[71]
Alginate	Flow-focusing	Ionic	General	[72]
Gelatin	Flow-focusing	Thermal	General	[73]
GelMA	Flow-focusing	Photochemical	General	[14,15]

## Data Availability

Not applicable.
